# Aggregation Analysis of Simulated Electric Field Poled Poly(methyl methacrylate) Doped with Tricyanopyrroline Chromophores

**DOI:** 10.1021/acs.jpcb.5c05224

**Published:** 2025-09-16

**Authors:** Nils M. Denda, Erik Rohloff, Peter Behrens, Andreas M. Schneider

**Affiliations:** † Cluster of Excellence PhoenixD (Photonics, Optics, and Engineering − Innovation Across Disciplines), 30167 Hannover, Germany; ‡ Institute of Inorganic Chemistry, 26555 Leibniz University Hannover, 30167 Hannover, Germany

## Abstract

Dipolar chromophore molecules embedded with noncentrosymmetric alignment in a polymer matrix may exhibit nonlinear electro-optical (EO) activity. The polymer matrix serves as a host, stabilizing the alignment of chromophores and conserving the EO activity in the glass state. However, at high chromophore number densities and/or elevated temperatures, aggregation may occur, resulting in a loss or at least altered EO response. Here, we present a novel and general Python-based tool for the analysis of aggregation and phase behavior. Our method provides frequency distributions of aggregate size (i.e., number of involved molecules) and aggregate types (i.e., the mutual molecular arrangement). The aggregation analysis is illustrated for the molecular dynamics simulation of electric field poling and relaxation (i.e., the analysis of the phase behavior). The analysis method helps to identify and visualize the process of aggregation and can be adapted to various models (e.g., liquid crystalline materials) if the shape of the molecule of interest is properly considered.

## Graphical abstract

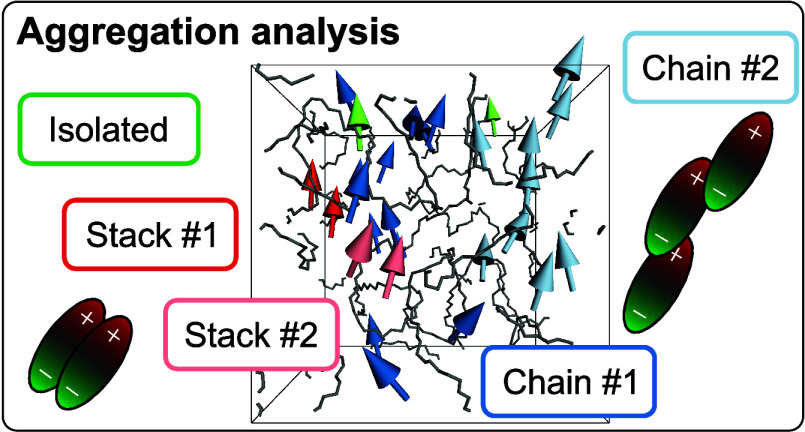

## Introduction

In this study, we examine the aggregation and phase behavior of chromophores (guests) embedded in a polymer matrix (host) for applications as an organic nonlinear optical (NLO) material. The chromophores have a donor–acceptor-structure with highly polarizable π electrons. If the dipoles are in noncentrosymmetrical order (e.g., after electric field poling), the material is electro-optic (EO) active and can show NLO effects, like the Pockels effect. Organic NLO materials have advantages regarding bandwidth, dielectric constant, half-wave voltage, EO coefficients, processability and material cost as compared to inorganic materials, e.g., lithium niobate.^1^ Future applications for organic NLO materials are emerging. There is a need for efficient high-bandwidth electrical-to-optical signal conversion over a broad range of frequencies (MHz–THz), combined with efficient, high bandwidth photo detection.^2^ Efficient signal conversion can be utilized for a wide variety of applications including telecommunication applications, detection, sensing, imaging, metrology, spectroscopy and super/quantum computing.^1,2^

For the host–guest materials discussed in this paper, a high chromophore density is desired to obtain a large EO effect. However, loadings beyond >25 wt % lead to lowered EO responses, which is often attributed to aggregation and/or phase separation,^1,3–7^ because chromophores are often strongly dipolar or zwitterionic and show poor solubility in low-polarity environments like polymers.^6^ When phase separation occurs, chromophores may reorient more easily, resulting in a loss of polar order and a loss or change in EO response.^6^ Elevated temperatures during manufacturing or in the application itself may also promote aggregation, resulting in a deterioration of the EO effect.^6^

Studies of the effects on the optical absorption of aggregated chromophores have a long history. First, additive behavior and electrostatic interactions were considered and gave satisfactory explanations for materials with small chromophore molecules and a medium or low chromophore content,^8^ because intermolecular interactions were approximately negligible.^9^ But in the last few decades, chromophores have been developed into multifunctional high-performance molecules with large π electron systems, dipole moments, and (hyper)­polarizability. These highly developed chromophore molecules may also have strong nonbonded interactions in aggregates, which have a profound effect on the EO activity and response.^9–11^ Deviations from additive superposition of EO molecular tensors become more important as the order of nonlinearity of the corresponding effect increases, due to collective, cooperative and responsive effects.^9–11^

On the experimental side, there are some well-established and long known strategies to deal with the “problem” of aggregation, e.g., covalent attachment of guest molecules in the backbone of the polymer or as side groups.^3^ However, covalent attachment also restricts chromophore mobility, which is detrimental to poling efficiency. Therefore, chromophores have been modified by introduction of large cyclic aromatic π electron systems to control aggregate formation or by introduction of alkyl groups to improve solubility.^6^ Bulky side groups at the chromophores can improve the reorientation in the poling field, enhance the poling efficiency and may reduce aggregation,^1^ but cannot prevent it completely.^6^ Nevertheless, the changed EO response of aggregated chromophores may also be exploited in applications. An ideal supramolecular arrangement (ideal aggregate) of chromophores could lead to a superlinear enhancement of the first hyperpolarizability (e.g., demonstrated in ref 9). Different types of aggregates can be also useful for complementary light absorption for better performance of solar cells based on organic polymers for photovoltaics.^12^

For experimental investigations of chromophore aggregates a peak fitting procedure of the UV/vis absorption spectra was established to identify isolated chromophores and chromophores in different types of aggregates.^13^ This experimental method proved to be beneficial in different studies of chromophores in polymer systems.^6,12,14^ One of the most recent studies investigated the effects of bulky groups at the chromophores on the aggregation behavior and could show that bulky groups at the chromophore have only a modest effect on reducing aggregation.^6^

First computational investigations of host–guest materials on the atomistic level were conducted by Kim and Hayden.^15^ Within their study, a radial distribution function (RDF) analysis method was introduced that provides insights into the local dynamics of the polymer chains. Moreover, chromophore and polymer matrix interactions (chromophore–side group/chromophore–backbone) were studied. The established method was applied many times so far.^16–20^ More recent studies added to this method counts of the noncovalent interactions (π–π interactions or H-bonds),^7,19,21–23^ an analysis of the relative alignment of aggregated chromophores^24^ or analysis of thermodynamic properties (free energy values for aggregate formation).^25^

In order to gain insight into the architecture and implications within complex hybrid materials, it is necessary to understand the aggregation and phase behavior. A comprehensive understanding of the orientation of the chromophore dipoles relative to each other, as well as the number of chromophores involved in one aggregate, is essentially required.^6,10,11,13^ However, aggregate sizes (i.e., number of involved chromophores in one aggregate), different aggregate types, and frequency distribution functions thereof are not directly identifiable solely based on RDF analyses. So far, to the best of our knowledge, there is no computational analysis method available that investigates the aggregation and phase behavior in detail (e.g., aggregate type and size distribution) as we propose to do.

Molecular Dynamics (MD) methods are characterized by their ability to produce absolute (countable/directly measurable) results under ideal controlled conditions. In addition, MD methods provide direct insights into the atomistic level. For example, applying our method, aggregates are identified, and their size and composition can be highlighted. However, direct comparisons between the simulation and experiment are always challenging. Regarding the aggregate analysis, only relative statements can be made in the experiment. For example, experimental statements can only be made about the proportions of active or inactive chromophores (see, for example, the absorption cross-section analysis described in ref 6), or changes can be measured, such as the change in electro-optical activity, as described in ref 6.

The electro-optic tensor element *r*_33_ is an important performance indicator, but it depends on several quantities, such as the order parameter ⟨cos^3^ θ⟩ and hyperpolarizability β of the chromophore molecules.^26^ If a change in the EO coefficient is experimentally measured, e.g., after a certain temperature program), then it is unclear what the change in the EO effect consists of. The change in the measurement signal can be caused by several factors, e.g., aggregation (attenuation or amplification of EO activity^9–11^ ), loss of orientation, or disintegration of the chromophores.^6^ Therefore, it is difficult or impossible to deduce absolute direct statements from spectroscopic methods. The MD methods we have developed can be used as tools to gain direct insights at the atomic level and to visualize or specifically test structure–property relationships, reducing the amount of effort spent in the laboratory.

## Computational Details

Force field (FF) calculations were conducted with BIOVIA Materials Studio 23.1 ^27^ utilizing the COMPASS III force field.^28^ The simulation protocol, computational details and results are presented in our previous paper.^26^ The present study relies on the formerly produced molecular dynamics (MD) simulation trajectories and provides in-depth phase behavior and aggregation analysis. In the following, the host–guest models and the cornerstones of the simulation protocol are briefly introduced.

The host–guest models are composed of three atactic poly­(methyl methacrylate) (PMMA) chains with 100 repeat units (RU) each and a variable number of chromophore molecules, which are placed into the cell with a Monte Carlo-based algorithm. The different model compositions are displayed in Table 1. Nine independent models were constructed for each chromophore mass concentration.

**Table 1:** Model Compositions (RU = Repeat Units of PMMA)^a^

Amount of C3 (wt %)	3 × 100 RU + #C3	#Atoms
10	7	4947
15	12	5262
20	16	5514
25	22	5892
30	28	6270

a Data reproduced from ref 26. Copyright 2025 The Authors.

The different stages of our previously developed simulation protocol^26^ is visualized in Figure 1. The structure model preparation consists of two stages: First, the packing of the chromophore and polymer molecules into the simulation cell is performed. The packing procedure is followed by an annealing stage (2) to equilibrate the models at the temperature of the electric field poling process (*T* = 450 K). The standard electric field poling process comprises two poling stages. The first poling stage (3) is performed at temperatures above the apparent glass transition temperature (*T*_g_) and the second poling stage (4) is conducted at room temperature (*T*_rt_ = 300 K). The last simulation stage (5) is carried out at elevated temperature but below the glass transition temperature.

**Figure 1:**
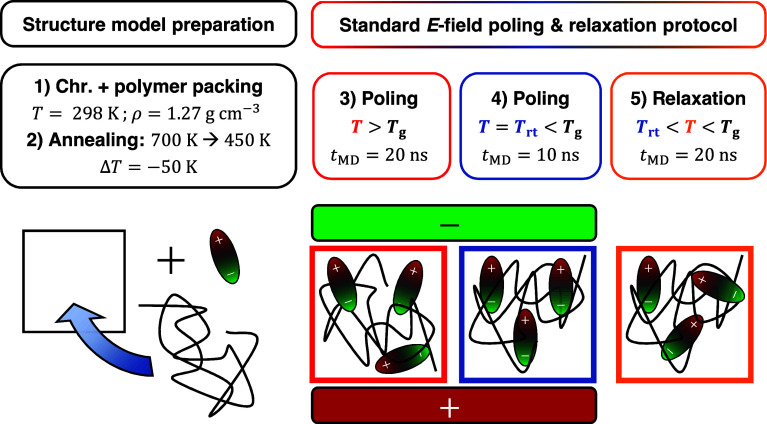
Illustration of our previously developed simulation protocol.^26^ The initial part encompasses the structure model preparation, which includes the chromophore (chr.) and polymer packing, followed by an annealing stage. The second part shows the standard electric field poling and temperature protocol with the corresponding molecular dynamics time frames (*t*_MD_). *T*_g_ = glass transition temperature, *T*_rt_ = 300 K.

In the first poling stage (stage 3), chromophore alignment is facilitated at elevated temperatures well above the *T*_g_. To conserve the chromophore alignment in the vitrified polymer host, the system is modeled subsequently at room temperature with an applied electric field (stage 4). The last stage was introduced to trigger reorientation and investigate the (long-term) stability of the chromophore alignment.

The order parameter ⟨cos^3^ θ⟩ represents the alignment of a collection of dipoles. The angle θ represents the angle between each molecular dipole moment and the poling field direction. We will use two terms to describe the behavior of the chromophores: Poling efficiency refers to the averaged order parameter value after the second poling step (stage 4) and alignment stability refers to the averaged order parameter after the relaxation step (stage 5).

### Chromophore Distribution and Aggregation

The main objective of this study is the investigation of the spatial distribution of the chromophore guests in the polymer host and their possible aggregation behavior. The chromophores have distinct properties compared with the polymer host. The functional groups of the chromophores, generating an electron donor–acceptor structure, may lead to aggregation caused by electrostatic interactions. To gain deeper insights into the host–guest models and enhance understanding of the phase behavior, a few selected models are analyzed in detail, and their phase behavior throughout the employed poling process is monitored.

The dimensions of the flat prolate chromophore C3 are visualized in Figure 2. Three characteristic coordinates are chosen to calculate chromophore pair distances and to evaluate these pair distances. One characteristic point is the so-called midpoint, located at the center of the geometry of each chromophore (represented by the pink point in the middle of C3 in Figure 2). Two other specific reference points are the head and tail atom, located on the nitrogen atom of the diethylamino group (red, right-hand side of the midpoint) and the carbon atom of the dicyanoethylene group (green, left-hand side of the midpoint), respectively.

**Figure 2:**
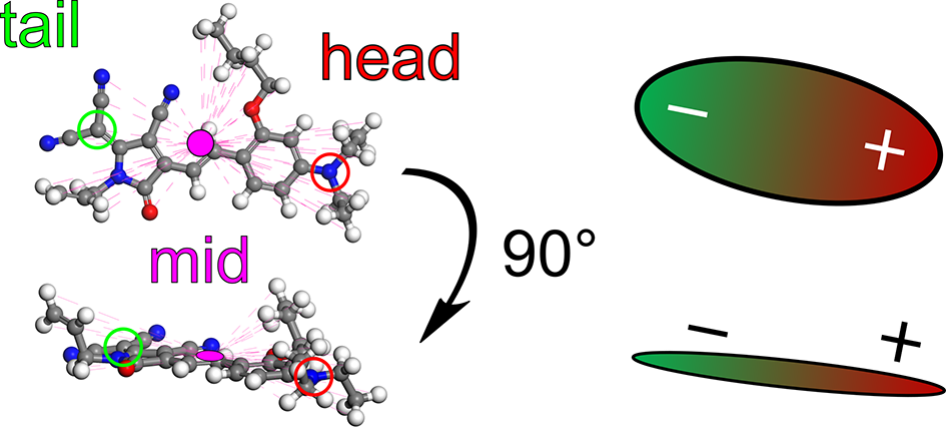
Atomistic structure of the flat-prolate chromophore C3 and representative visualization.

The aggregation behavior of the chromophores was analyzed by using a custom Python script developed for this study. The input, output and operating principle of the tool are outlined in the Supporting Information, and the tool is available at https://gitlab.uni-hannover.de/erik.rohloff/aggregation-analysis/.

## Results and Discussion

First of all, a short overview is provided regarding poling efficiency and alignment stability, for a deeper discussion we refer to our previous paper.^26^

Subsequently, the aggregation and phase behavior of the chromophores are analyzed and discussed, and an overview of the steps is displayed in Figure 3. First, the aggregation class analysis of entire model sets are presented and discussed. Isolated and aggregated chromophores are identified, and the class of aggregation is determined. The degree of aggregation (DOA) is obtained (relative proportion of aggregated chromophores). Next, the size distribution of aggregates is analyzed and presented. The relative amount of chromophores participating in an aggregate is plotted against the aggregate size. Finally, a few selected models are analyzed in more detail. First, the aggregation behavior is analyzed in the course of the simulation protocol (3a) and subsequently the order parameter is examined of isolated and aggregated chromophores (3b).

**Figure 3:**
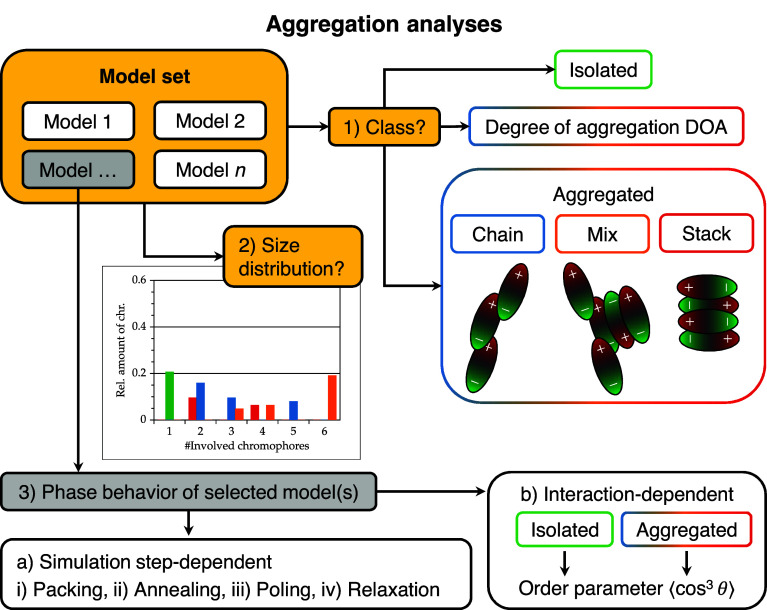
Overview chart of the performed analyses. The first two analysis steps are performed on entire model sets (here: *n* = 9). The total number of chromophores *N*_chr_ depends on the model set concentration ranging from 10 to 30 wt %: *N*_chr_ = *n* × (7 ... 28 chromophores) = 63 ... 252 chromophores.

The Supporting Information provides all graphs about aggregate class and aggregate size distributions for each individual model at each of the three model set concentrations ranging from 10 to 30 wt %.

### Order Parameter, Poling and Relaxation

Figure 4 shows the order parameter ⟨cos^3^ θ⟩ of the standard poling and relaxation simulation protocol. Order parameter values are averaged over 2 ns for each model of a model set to ensure a reliable estimate. The order parameter values presented in Table 2 are average values of nine independent models representing a model set for one specific chromophore concentration. The uncertainties are calculated based on the standard deviations of the nine independent models to take structural variability into account. Increased chromophore content leads to slightly larger order parameter values, but if model standard deviations are included, then the differences are not significant. Applying strong electric fields in the manufacturing process, a poling efficiency of around 90% can be reached, and the alignment stability at ambient conditions (room temperature) is expected to be approximately 75%.

**Figure 4:**
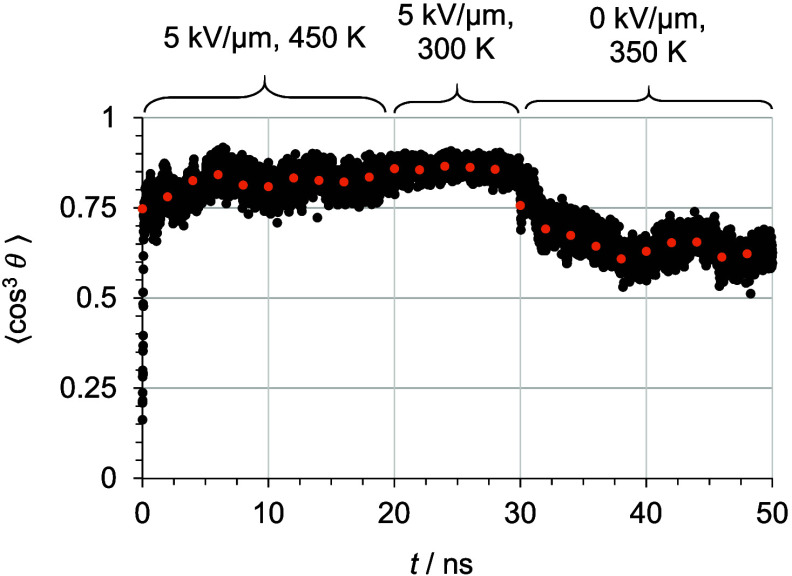
Order parameter ⟨cos^3^ θ⟩ during and after the standard electric-field poling process of one example structure (10 wt % C3 in PMMA; orange bullets, 2 ns averages; black bullets, order parameter of every single frame). The average value at 28 ns is referred to as poling efficiency, and the last average value at 48 ns is referred to as alignment stability. Reproduced from ref 26. Copyright 2025 The Authors.

**Table 2:** Average Order Parameter Values ⟨cos^3^ *θ*⟩^a^

	⟨cos^3^ θ⟩	
Model set^b^	after poling	after relax
10 wt %	0.87 ± 0.05	0.70 ± 0.06
15 wt %	0.88 ± 0.02	0.72 ± 0.05
20 wt %	0.91 ± 0.02	0.72 ± 0.04
25 wt %	0.90 ± 0.01	0.75 ± 0.03
30 wt %	0.92 ± 0.02	0.75 ± 0.03

a Data reproduced from ref 26. Copyright 2025 The Authors.

b Each model set consists of nine independent models.

Figure 5 shows order parameter values in the course of long-term relaxation simulations at low and high chromophore concentration and at temperatures below and above *T*_g_. Simulations with temperatures below *T*_g_ prove alignment stability at approximately 75%, whereas temperatures above *T*_g_ reveal a drop in the order parameter. The order parameter of both high-temperature simulation models (with low and high chromophore concentration) drop to approximately 30%. The phase behavior of each model is analyzed and discussed in detail later, but the order parameter in the course of the high-temperature relaxation already shows distinct phase behavior: The order parameter value of the low-concentration model moves around 50%, whereas the order parameter value of the high-concentration model decreases continuously to a lower level. The fluctuation of the order parameter of the low-concentration model arises from movable isolated chromophores, whereas chromophores in high-loaded models are reorientating collectively and therefore more slowly.

**Figure 5:**
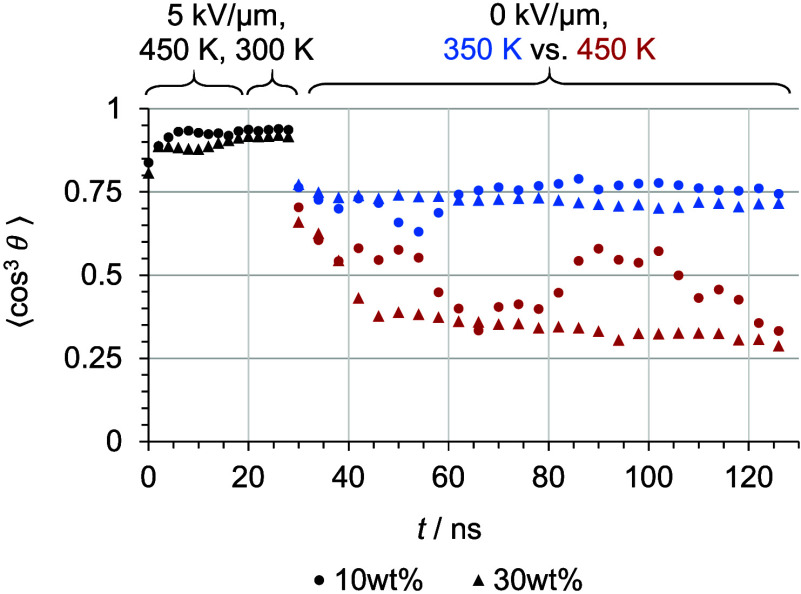
Order parameter ⟨cos^3^ θ⟩ during long-term relaxation simulations at temperatures below the estimated glass transition (blue) and above this value (red) at the lowest (dots) and highest (triangles) weight percentages of C3 in PMMA. Reproduced from ref 26. Copyright 2025 The Authors.

### Definition of Separation Distances for Aggregation Analysis

Figure 6 shows the statistical analysis of the *k*-smallest pair distances in an isotropic condensed phase of pure chromophore C3. There are two main separation distances of importance (compare Figure 2 in the computational methods section), i.e., the head-to-tail (HT) or the midpoint-midpoint (MM) distance. The error bars represent the standard deviation. The minimum and maximum values of each pair distance highlight the large spread of observed values, arising from the anisotropic shape of the chromophore molecule (flat prolate spheroid with a sterically demanding side chain). The explicit values of Figure 6 are provided in Table 3. In the following, the average pair distance plus one standard deviation is set as the separation distance to identify an aggregated pair of chromophores.

**Figure 6:**
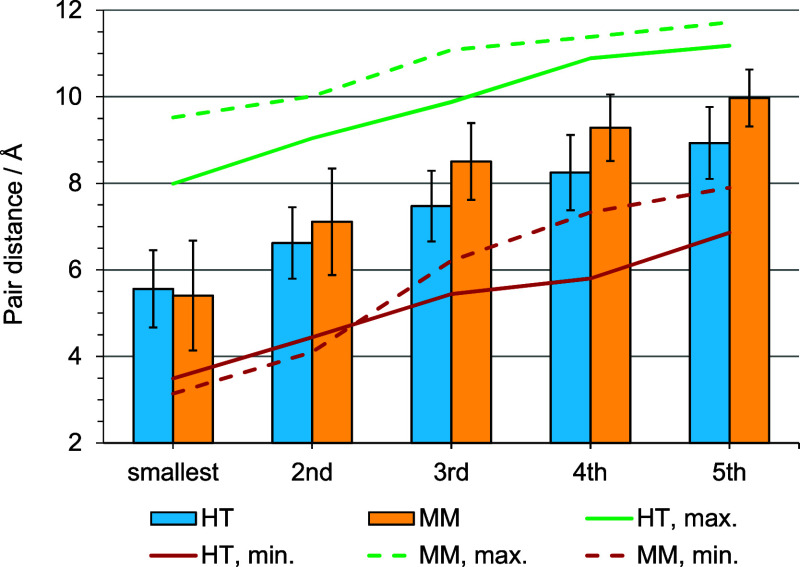
Minimum, average and maximum values of *k*-smallest pair distances in a condensed phase of 300 C3 chromophore molecules at 300 K (isotropic phase). Error bars represent the standard deviation. HT: head-to-tail distances. MM: midpoint-midpoint distances. The model was annealed and equilibrated in a temperature range from 700 to 300 K in 50 K steps, in the *NpT* ensemble (for details refer to our previous paper^26^).

**Table 3:** Pair Distances of a Condensed (Isotropic) Phase of 300 C3 Chromophore Molecules at 300 K^a^

	head-to-tail distances/Å				
	smallest	second	third	fourth	fifth
Min.	3.5	4.4	5.4	5.8	6.9
Max.	8.0	9.0	9.9	10.9	11.2
Average μ	5.6	6.6	7.5	8.2	8.9
Std. dev. σ	0.9	0.8	0.8	0.9	0.8
μ + σ	6.5	7.4	8.3	9.1	9.8
μ + 2σ	7.3	8.3	9.1	10.0	10.6

	midpoint–midpoint distances/Å				
	smallest	second	third	fourth	fifth
Min.	3.1	4.1	6.2	7.3	7.9
Max.	9.5	10.0	11.1	11.4	11.7
Average μ	5.4	7.1	8.5	9.3	10.0
Std. dev. σ	1.3	1.2	0.9	0.8	0.7
μ + σ	6.7	8.3	9.4	10.1	10.6
μ + 2σ	7.9	9.6	10.3	10.8	11.3

a The model was annealed and equilibrated in a temperature range from 700 to 300 K in 50 K steps, in the *NpT* ensemble (for details refer to our previous paper^26^).

The selected characteristic points (head, mid, tail), from which distance criteria (minimum pair distances of the aggregation) were derived, depend on the geometry of the objects under investigation (here: chromophores). In its isolated state, the chromophore has an extended π electron system that tends to adopt a planar structure. We defined three characteristic points that are mapped onto rigid structural elements of the chromophore. These points are therefore assumed to be localized, i.e., constant in their relative local position.

The distance criteria depend on which reference system is selected. We selected a fluid, pure chromophore phase to observe the actual shortest pair distances in the ensemble of chromophores and chose these as the basis for the evaluation. To take structural variations and slight flexibility into account, we evaluated the average value plus the single standard deviation of the 300 shortest chromophore pair distances. We consider it necessary to add the standard deviation because the ethyl groups at the head of the chromophore are quite flexible, and the entire chromophore can be easily bent or twisted in the polymer host by external forces.

The selected distance criteria and the sensitivity to aggregation events always form a mutually interrelated system (selected criteria ↔ observable aggregation events). The following examples (a vs b) may illustrate this point: (a) The minimum distance criterion could be chosen very strictly (short). For example, the densest-packed chromophore pair without averaging over an ensemble could be chosen as a reference to define the minimum distance criterion for the aggregation. However, there is a risk that only a few or specific signals (= aggregation events) are observed (very sensitive result), which does not correspond to reality, since chromophores already interact with each other, e.g., via van der Waals interactions, or are present in pairs or groups in a polymer pocket. (b) In contrast, if the minimum aggregation pair distance criterion is chosen too large, one would incorrectly observe that most chromophores are aggregated (false-positive result, insufficient sensitivity), since the chromophores are either too far apart to interact or there are even other molecules (e.g., the polymer chain) between the supposedly aggregated chromophores.

### Aggregation Class Analysis

Figure 7 shows one model of the 30 wt % model set after relaxation as an example. Dipole moments are colored to visualize the different aggregates and aggregate classes. Note that periodic boundary conditions are considered in the aggregation analysis. The three uppermost dark blue dipole moments are periodic images of the three lowest dark blue colored dipoles. There are two chain-like aggregates (light blue/dark blue), two stacked pairs of chromophores (red), and two isolated chromophores (green). The power of the developed analysis method lies in the identification of independent aggregates and the subsequent statistical plot output, which simultaneously provides profound insights into the phase behavior and aggregation dynamics of many simulation models. In the following, statistical plots of entire model sets are in focus, and subsequently, a few selected models are discussed in more detail.

**Figure 7:**
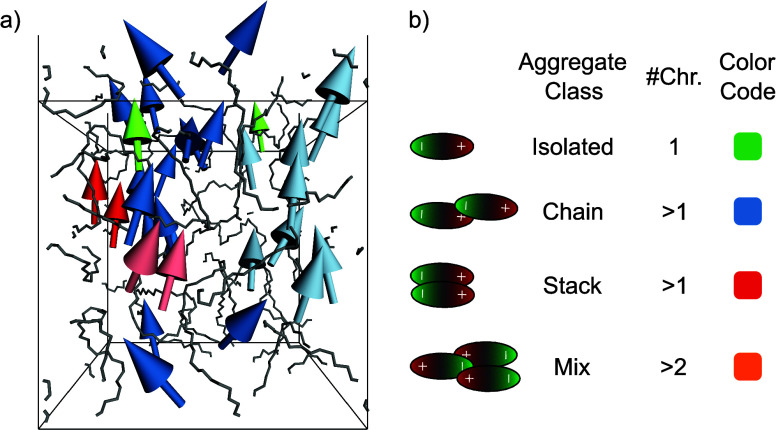
(a) An exemplary picture of the individual dipole moments to visualize the different aggregate classes after relaxation of one model of the 30 wt % model set. The same color indicates that the chromophores belong to the same aggregate. (b) Quick reference for an overview of the different aggregate classes and applied color code in the following diagrams. #Chr. = number of chromophores.

Figure 8 shows, on average, observed aggregate classes in the course of the standard poling and relaxation simulation protocol. One model set consists of nine independent models to take into account the variability of amorphous polymer structures. In the following, the term “degree of aggregation” (DOA) is introduced to describe the relative amount of chromophores which are incorporated in aggregates (the relative amount of the three different aggregate classes are cumulated). Independent of the chromophore concentration, the packing process produces more aggregates than in the remaining simulation steps. The DOA after packing is above 80% for the model containing 10 and 20 wt % and around 90% for the model containing 30 wt %. After all remaining simulation steps, the DOA is constant and located at approximately 50% for low concentration models, 70% for 20 wt % models and 80% for high concentration models. This observation shows the importance of the annealing for structure preparation. The Monte Carlo algorithm is able to generate varying amorphous start structures. However, it is computationally demanding to place large chromophore molecules between polymer chains or to grow polymer chains around these large, sterically demanding molecules. Some chromophore molecules are more closely packed than in an equilibrated host–guest model.

**Figure 8:**
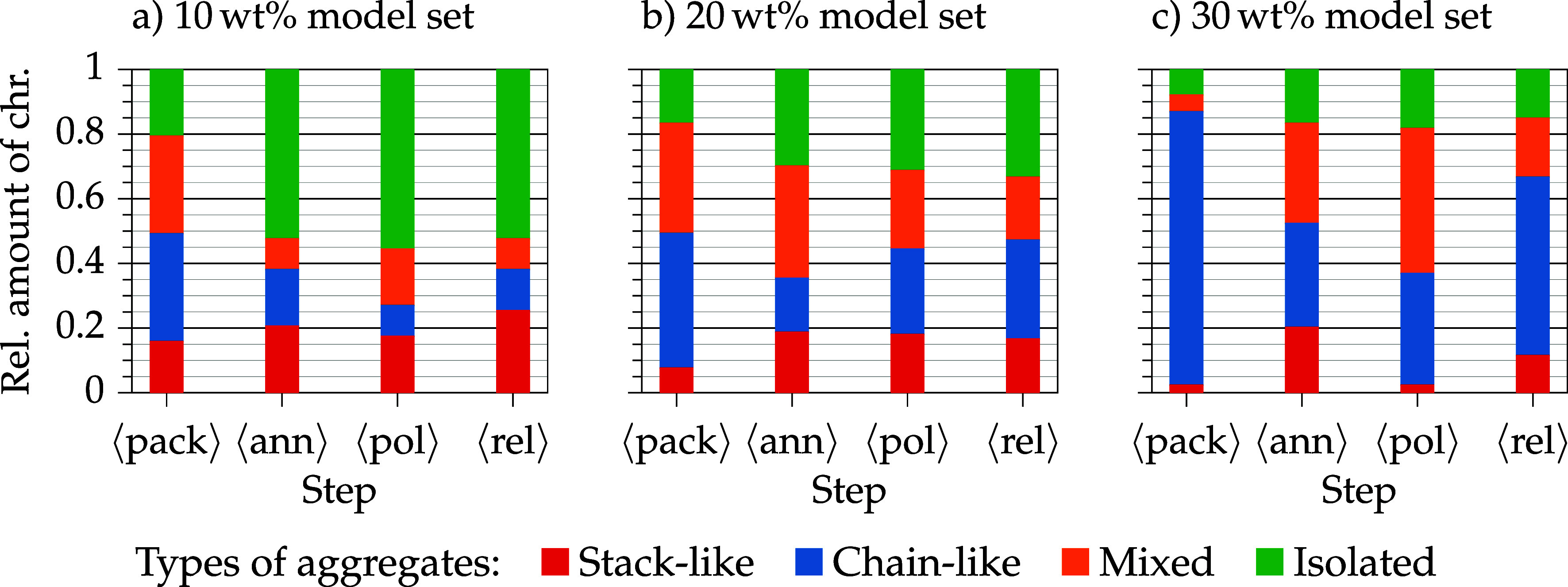
Observed aggregates on average ⟨...⟩, dependent on the model set concentration (a–c) in the course of the standard poling and relaxation protocol: after packing (pack) → annealing (ann) → poling (pol) → relaxation (rel). One model set consists of nine models.

In general, in low concentration models (10 and 20 wt %) a larger amount of stacked aggregates is observed than in the highest loaded models. As the loading of chromophores increases, the amount of chain-like aggregated chromophores also rises. This observation is explained by the flat-prolate shape of the chromophores. Especially at high concentrations, chromophores may form strands in between the polymer strands and this effect is even more pronounced after poling and relaxation (see Figure 8c).

The 20 wt % model set has an average DOA of 70% and the type of aggregation is rather evenly distributed across all aggregation classes (after annealing and throughout the remainder of the simulation steps). The amount of stacked chromophores is roughly constant after annealing, and the number of chain-like aggregated chromophores is slightly higher, whereas the amount of mixed aggregated chromophores is lower.

The poling process of the 30 wt % models leads to a decrease of stacked aggregates and a rise of mixed aggregates. High temperatures and strong electric fields enhance and promote rearrangement of chromophores so that stacks are broken up. Because of the prolate chromophore shape and the polymer strands, chromophores are also arranged in strands. The strong poling field may also affect the low-polarizable polymer host, so that polymer strands may perturb “ideal” packing of chromophores (= stack or chain) leading to mixed aggregates (compare order parameter values: after poling all around 90%, see Table 2). After the electric field is turned off, stress in the host–guest systems can be released, so that some stacked aggregates may (re)­arrange in the relaxation step. Mixed aggregated chromophores may rearrange to chains driven by electrostatic forces when no poling field is present.

Figure 9 contrasts the low concentration model set with the high concentration model set. Additionally, the bar plots of the long-term relaxation simulations are shown. The models for the long-term relaxation (LTR) simulations were chosen according to their order parameter value. These two models have a median order parameter value after the standard relaxation step. The model set averages (first four bars) have been discussed before and are only provided to relate them to the single model values after standard relaxation and LTR. The low concentration model chosen for LTR has a DOA above average (+35%) and only consists of stacked aggregates. The DOA of the low concentration model is comparable to the DOA of the high concentration model, which consists of only chain and mix aggregates, roughly representing the average behavior of the model set. In the course of the LTR below *T*_g_ (LTRb), the DOA of the low concentration model decreases by half, while the high concentration model remains at the same DOA. The low concentration model has a DOA within the expected range after LTRb compared to the model set average after standard relaxation, but is still only composed of stack aggregates. The high concentration model contains chain aggregates after LTRb and also roughly fits to the model set average after standard relaxation, when uncertainties are taken into account (uncertainties for all models can be assumed to be approximately 10%).

**Figure 9:**
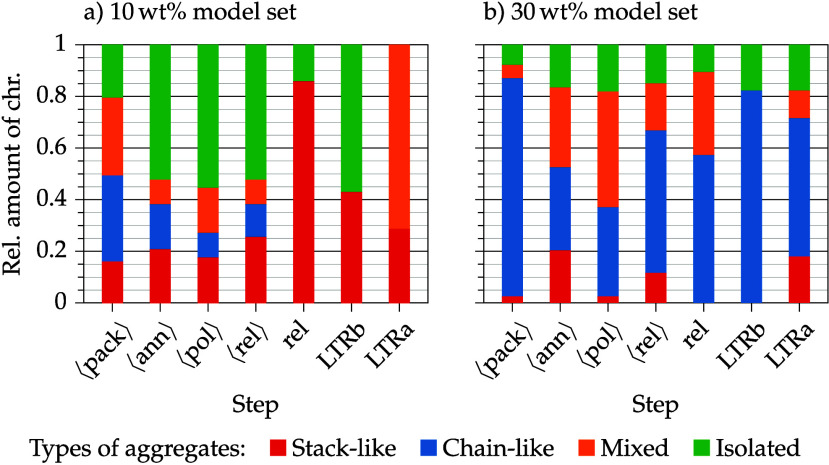
Observed aggregates in 10 (a) and 30 wt % (b) model sets in the course of the standard poling and relaxation program (after packing (pack) → annealing (ann) → poling (pol) → relaxation (rel), *t*_relax_ = 20 ns) in comparison to the long-term relaxation experiments (*t*_relax_ = 100 ns) below (LTRb) and above (LTRa) the glass transition *T*_g_. Averages over nine independent models are denoted by brackets ⟨...⟩.

The LTRb is conducted at 350 K with no electric field so that the overall molecular mobility is slightly enhanced compared to room temperature and the host–guest system can release stress caused by the strong poling field (light annealing conditions). These LTRb conditions promote polymer chain relaxation, especially during the extended simulation time. At low chromophore concentration with previously stacked aggregates, polymer chains may rearrange, break up stacks, and isolate chromophores. In contrast, at a high chromophore concentration with an excess amount of chain aggregates and a few mixed aggregates, the chromophores of these mixed aggregates may realign, driven by electrostatic forces of neighboring chromophore molecules. Consequently larger and/or more chain-like aggregates are formed.

The LTRb simulation result confirms the average aggregation result of the model set after the standard relaxation with a shorter simulation time. This holds especially true for the low concentration model, which seems to be an outlier regarding aggregation after standard relaxation compared with the whole model set. Computational effort can be lowered and structural variability can be represented, when multiple models are calculated in parallel and subsequently results are averaged (exemplary computation times of standard vs long-term relaxation are added in the Supporting Information).

The long-term relaxation above *T*_g_ (LTRa) is performed at 450 K with no electric field. Not only is the chromophore mobility enhanced but polymer chain mobility is also facilitated. Full aggregation can be observed for the low concentration model, but stacking is decreased compared to the other relaxation protocols. Concluding these observations, isolation of chromophores by the polymer host and the preservation of the well-aligned state are temperature sensitive and critical for optical activity. The high concentration model shows different behavior: The DOA is comparable to the standard relaxation program; stacking is slightly increased, whereas mixed aggregates and chain-like aggregates are slightly decreased in their occurrence. The high amount of sterically demanding chromophores between the polymer chains likely prevents chromophore movement across the polymer host and hinders further aggregation (from 90% to 100%). The chromophores are organized in multiple mixed aggregates in which most of them are parallel to each other. Single chromophores in these aggregates may realign, so that the aggregation class changes. This results in either chain- or stack-like aggregates, and thus, a smaller amount of mixed aggregates remain. The amount of stacked aggregates is comparable to the model set average after standard relaxation (if uncertainties of approximately 10% are taken into account).

### Aggregate Class and Size Distribution Analysis

Figure 10 and Figure 11 show the average chromophore size and class distribution in the course of the standard poling and relaxation protocol dependent on the model set concentration (10 and 30 wt %). The analyses were performed on the entire model sets. Each model set contains nine models (*n*_models_ = 9). The relative amounts are related to the populations of the chromophores of the whole model sets, i.e., *N*_tot_^chr^ = *n*_models_ × 7 = 63 for the 10 wt % model set and *N*_tot_^chr^ = *n*_models_ × 28 = 252 for the 30 wt % model set, respectively.

**Figure 10:**
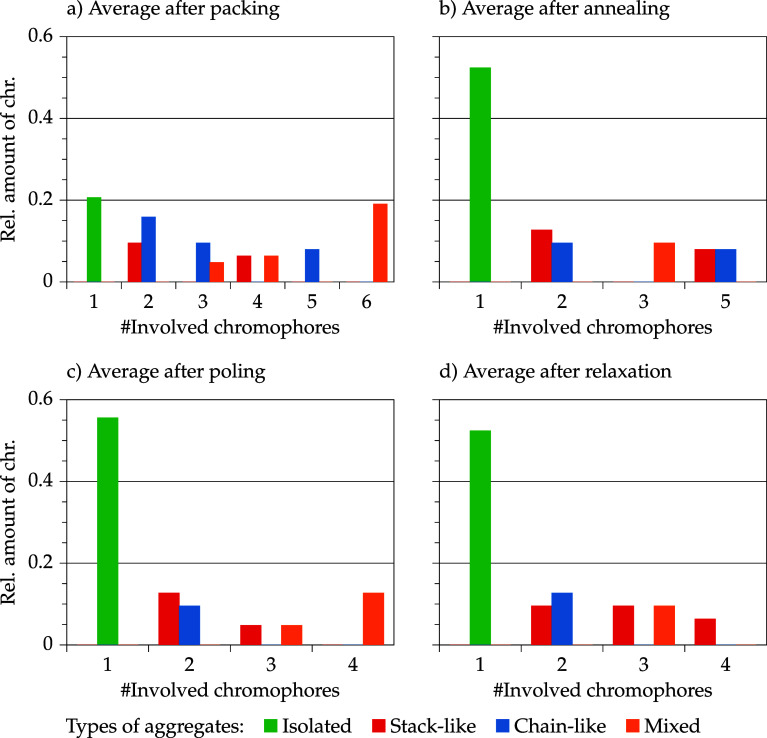
Average aggregate size distribution and average aggregation class distribution in the course of the simulation protocol (a–d) of the 10 wt % model set: *N*_tot_^chr^ = 63.

**Figure 11:**
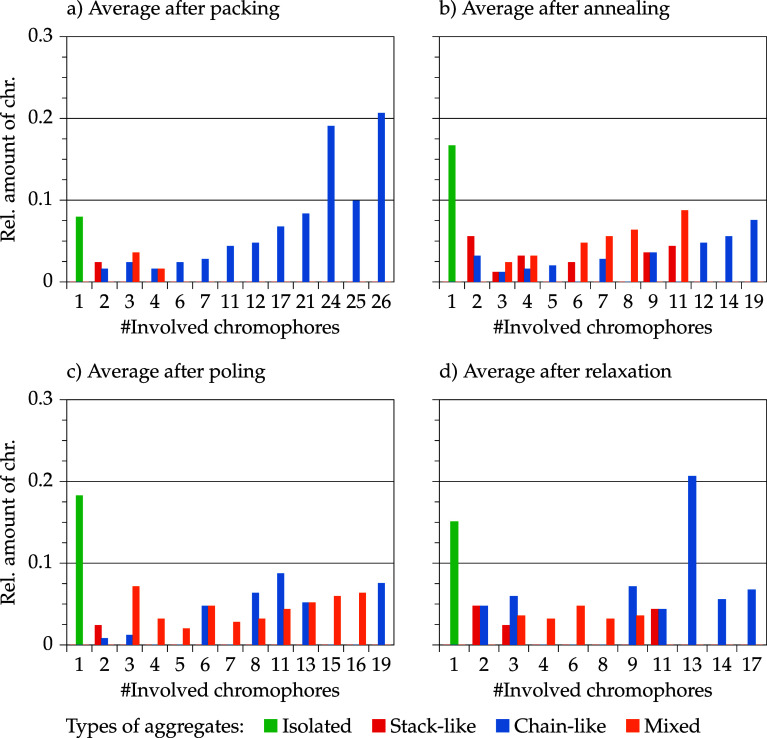
Average aggregate size distribution and average aggregation class distribution in the course of the simulation protocol (a–d) of the 30 wt % model set: *N*_tot_^chr^ = 252.

At low concentrations, only small aggregates are present, and in the course of the simulation, the chromophores are mainly separated and isolation increases. After annealing and further simulation steps, the amount of isolated chromophores is disproportionately larger compared with the packing step. For the low concentration models, isolated chromophores are more frequently observed than any other aggregate class. After packing and poling, larger aggregates (≥3) are primarily mixed. After relaxation, larger aggregates are stacked. Chain-like aggregates are only present as pairs after poling and after relaxation. In conclusion, 10 wt % models enable a homogeneous distribution of isolated chromophores in the polymer host, and only a few, small aggregates are present, which are most likely stacked or mixed. The order parameter of isolated and aggregated chromophores is analyzed after discussion of the average size and class distribution of the high concentration model set.

At high concentrations (Figure 11), primarily large chain aggregates are generated by the packing process and are then randomized through annealing. After annealing, large and very large aggregates (>10 and >20) are broken up. The amount of isolated chromophores is more than doubled. In the midsize range (4 to 11 aggregated chromophores) mixed and stack-like aggregates arise. A less skewed size distribution is yielded by the annealing process.

The poling merely influences the amount of isolated chromophores. Stacks are broken up and are rarely visible. The size distribution of the aggregates is not strongly affected by the poling process but leads to an excess of mixed aggregates.

With relaxation, the amount of isolated chromophores is slightly decreased and at large aggregate sizes (>10) chains are re-established, whereas mixed aggregates are only visible in the midsize range (3–9). Prealigned aggregates (during annealing: chains or stacks) are impeded by polymer chains which react to the strong electric field or which are also pushed away because of neighboring chromophores reacting to the strong field. The relaxation process probably releases strain, so that large aggregates (>10) may realign and form chains. Midsized aggregates cannot realign collectively without an external field and the sterically demanding chromophores mainly remain as mixed aggregate. Only a few chromophores, presumably isolated or detached chromophores, are able to move to an aggregate (decrease of isolated chromophores) or are able to realign forming small stack aggregates.

### Simulation Step-Dependent Phase Behavior Analysis

Figure 12 and Figure 13 show the aggregate class and aggregate size distribution of selected models of the 10 and 30 wt % model set. The models were selected according to their order parameter value after relaxation. No clear conclusions for the 10 wt % models can be made after packing and after annealing (= before poling). It is notable that chromophores in models with median and low order parameter values are incorporated in larger mixed aggregates (3–4 chromophores) after poling, whereas the model with the highest order parameter value has a pair of stack and a pair of a chain aggregates. After relaxation, the model with the highest order parameter value has also the highest amount of isolated chromophores and only one pair of stacked chromophores. The other two models are characterized by larger aggregates and a higher DOA. Intermolecular interactions are dominant for these two models. This aggregation has an impact on the overall chromophore behavior (interdependent realignment) and affects the model order parameter. Moreover, it is remarkable that models with higher average order parameter values are more likely aggregated in a stack-like manner, whereas the model with the lowest order parameter value consists of chain and mixed aggregates. This observation illustrates a stabilizing effect of stack-like aggregated chromophores. The explicit order parameter values for isolated and aggregated chromophores are discussed later on.

**Figure 12:**
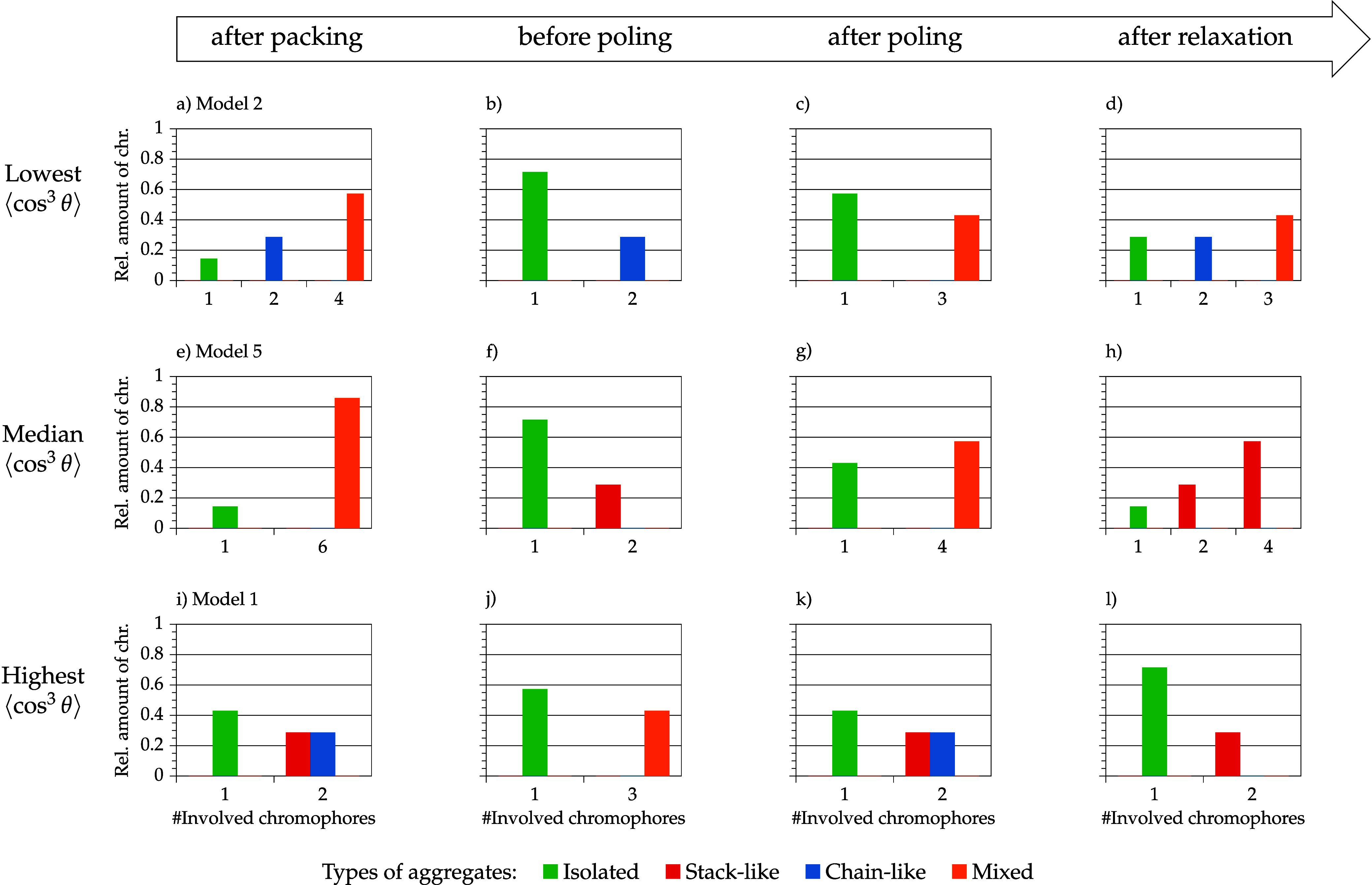
Selected models of the 10 wt % model set depending on their order parameter ⟨cos^3^ θ⟩ (model 2: (a–d)/model 5: (e–h)/model 1: (i–l)) and the corresponding aggregate size distribution and aggregate class distribution in the course of the simulation protocol (packing → relaxation).

**Figure 13:**
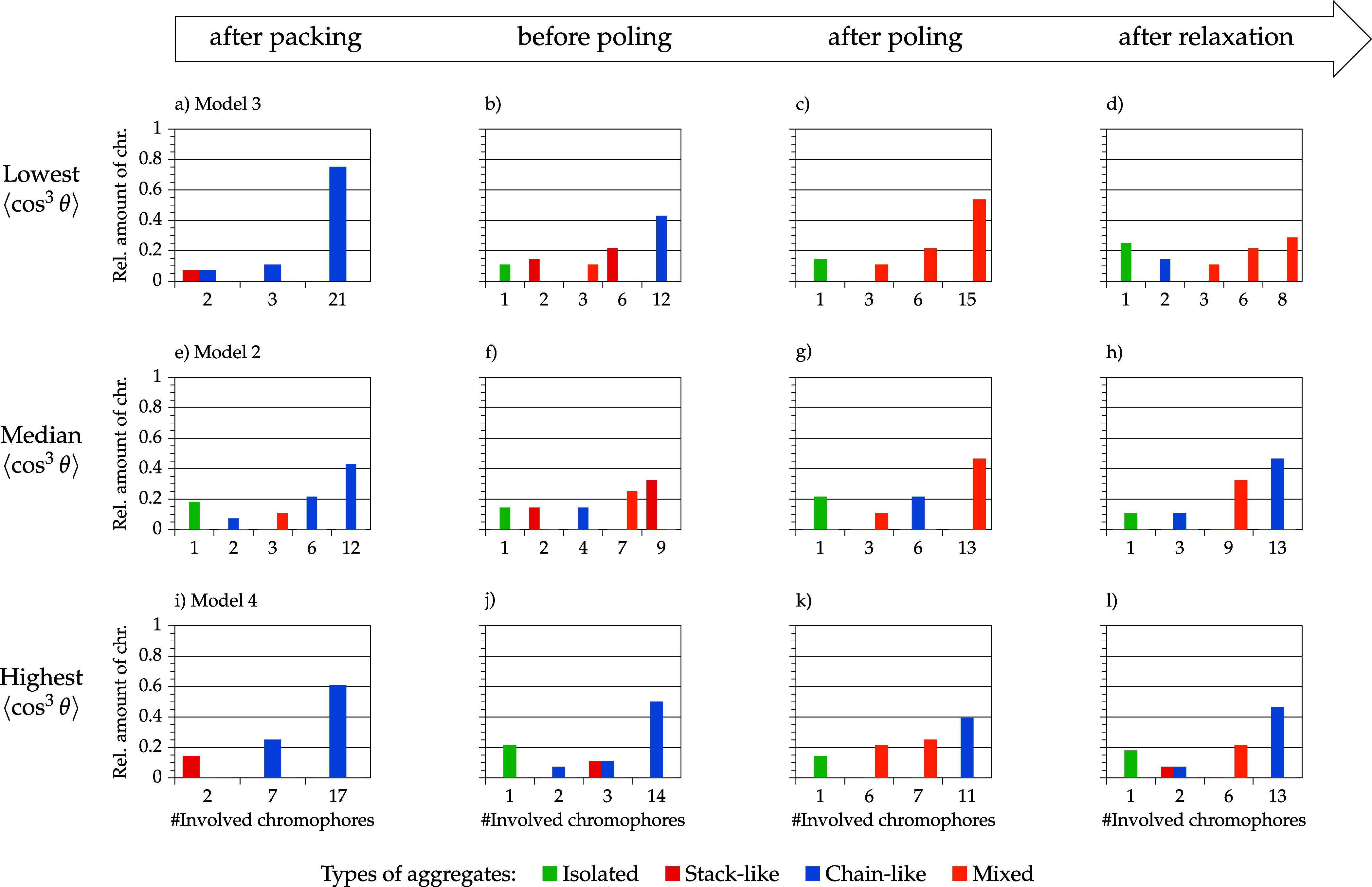
Selected models of the 30 wt % model set depending on their order parameter ⟨cos^3^ θ⟩ (model 3: (a–d)/model 2: (e–h)/model 4: (i–l)) and the corresponding aggregate size distribution and aggregate class distribution in the course of the simulation protocol (packing → relaxation).

For the 30 wt % model set before poling, it is noteworthy that the model with the highest order parameter value after relaxation contains one large aggregate, whereas the remaining chromophores are isolated or form only small aggregates. Both models with lower order parameter values contain more aggregates in the midsize range 3 to 10 and thus less isolated chromophores. This initial situation for poling affects the rest of the simulation results. Both models with lower order parameter values are dominated by mixed aggregates after poling, whereas the model with a higher order parameter value contains a large chain aggregate. In the relaxation step, the model with the highest order parameter value can preserve chromophore alignment with the large chain aggregate, whereas the two other models have smaller order parameter values because smaller mixed aggregates (or isolated molecules) are rearranging independently leading to an overall deteriorated order parameter value.

In conclusion, the analysis of the phase behavior results in two main insights: First, the type of aggregation is important and second, the size distribution of aggregates is significant for the alignment stability of chromophores. In the low-concentration model set, either isolated chromophores are able to reach high order parameter values (less chromophore–chromophore interactions) or stack-like aggregated chromophores have an good alignment stability. Highly loaded models demonstrate the impact of aggregate size: Small (mixed) aggregates (3–10 chromophores) show a poor alignment stability, whereas for highly loaded models large chain-like aggregates (>10 chromophores) point out a better alignment stability.

Finally, the results of the long-term relaxation simulations are discussed. Afterward, order parameter values of isolated and aggregated chromophores are presented. Figure 14 shows the aggregation class and aggregation size distribution of the standard and long-term relaxation simulation for one model at 10 wt % and for one model at 30 wt %. The low concentration model shows five isolated chromophores and one stacked aggregate of three after LTRb. At a temperature above *T*_g_, chromophores tend to aggregate in a disordered way, forming one large mixed aggregate of five chromophores and a stacked pair (DOA = 1). The high concentration model exhibits only chain-like aggregated chromophores and five isolated chromophores after LTRb. After LTRa more and smaller aggregates are formed. The DOA does not differ for both LTR protocols at high chromophore concentrations; presumably at high concentrations not all chromophores are necessarily involved in aggregates. This observation perhaps characterizes an equilibrium distribution.

**Figure 14:**
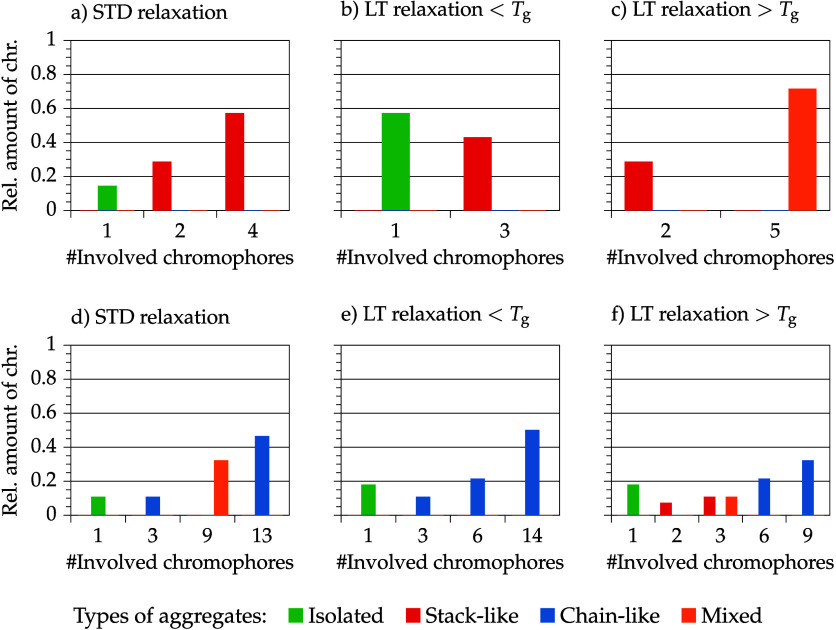
Aggregate size distribution and aggregation class distribution in the course of different relaxation protocols (standard (STD)/long-term (LT)) of a model with 10 wt % C3 (a–c): *N*_tot_^chr^ = 1 × 7 = 7 and of a model with 30 wt % C3 (d–f): *N*_tot_^chr^ = 1 × 28 = 28.

Polymer chain mobility shows distinct results on longer time scales for both concentrations: At low temperatures, large aggregates are stabilized in the polymer matrix. Only small changes in aggregate compositions after poling occur (compare Figure 12g and Figure 13g with Figure 14b,e). At temperatures above *T*_g_, aggregates are less cohesive; presumably, the overall enhanced mobility promotes rearrangements of polymer chains and embedded chromophores. For the low concentration model, chromophores are rearranging to larger aggregates and in the high concentration model (very) large aggregates are decomposing and rearranging to different midsize aggregates.

### Interaction-Dependent Phase Behavior Analysis

Table 4 and Table 5 show order parameter values ⟨cos^3^ θ⟩ of the selected few special models discussed above in more detail. These special models were chosen according to their order parameter averaged over 2 ns in the standard relaxation step. The order parameter values presented in Table 4 and Table 5 are order parameter values calculated of a single frame and not averaged over time, to supply insights of the orientation of isolated and aggregated chromophores, respectively. The so-called “total” order parameter is the order parameter of all chromophores in the snapshot. The order parameter values discussed here are not averaged over time, because aggregate compositions may change and inconsistencies would be inevitable.

**Table 4:** Order Parameter ⟨cos^3^ *θ*⟩ for Selected Models of the 10 wt % Model Set

		After poling		After relax		LTR^c^ < *T*_g_		LTR > *T*_g_	
Model	Group of chr^a^	DOA^b^	⟨cos^3^ *θ*⟩	DOA	⟨cos^3^ *θ*⟩	DOA	⟨cos^3^ *θ*⟩	DOA	⟨cos^3^ *θ*⟩
Lowest ⟨cos^3^ *θ*⟩	iso		0.81		0.64				
	aggr	0.43	0.61	0.71	0.48				
	tot		0.72		0.53				
									
Median ⟨cos^3^ *θ*⟩	iso		0.87		0.97		0.83		–
	aggr	0.57	0.91	0.86	0.75	0.57	0.82	1.00	0.42
	tot		0.90		0.77		0.82		0.42
									
Highest ⟨cos^3^ *θ*⟩	iso		0.88		0.75				
	aggr	0.57	0.74	0.29	0.87				
	tot		0.79		0.77				

a Group of chromophores: iso = isolated, aggr = aggregated, tot = total.

b DOA = Degree of aggregation = #(aggr chr)/#(tot chr).

c LTR = Long-term relaxation, only conducted for models with median ⟨cos^3^ θ⟩.

**Table 5:** Order Parameter ⟨cos^3^ *θ*⟩ for Selected Models of the 30 wt % Model Set

		After poling		After relax		LTR^c^ < *T*_g_		LTR > *T*_g_	
Model	Group of chr^a^	DOA^b^	⟨cos^3^ *θ*⟩	DOA	⟨cos^3^ *θ*⟩	DOA	⟨cos^3^ *θ*⟩	DOA	⟨cos^3^ *θ*⟩
Lowest ⟨cos^3^ *θ*⟩	iso		0.72		0.73				
	aggr	0.86	0.91	0.75	0.69				
	tot		0.88		0.70				
									
Median ⟨cos^3^ *θ*⟩	iso		0.82		0.72		0.76		0.34
	aggr	0.79	0.85	0.89	0.74	0.82	0.68	0.79	0.28
	tot		0.84		0.74		0.69		0.29
									
Highest ⟨cos^3^ *θ*⟩	iso		0.70		0.57				
	aggr	0.86	0.89	0.82	0.82				
	tot		0.86		0.78				

a Group of chromophores: iso = isolated, aggr = aggregated, tot = total.

b DOA = Degree of aggregation = #(aggr chr)/#(tot chr).

c LTR = Long-term relaxation, only conducted for models with median ⟨cos^3^ θ⟩.

Starting with the low concentration model with the lowest time averaged order parameter value, the total order parameter value of the snapshot is already the smallest after poling (0.72) and decreases for both aggregated and isolated chromophores, leading to a very low total order parameter after relaxation (0.53). The order parameter values of the other two models are significantly higher in total after poling and do not decrease as drastically as those for the model discussed first. The model with median order parameter has one well aligned chromophore (0.97) and aggregated chromophores which are fairly well aligned (0.74) in the relaxation snapshot. The model with the highest order parameter shows a decrease in DOA (in contrast to the aforementioned models), and both groups of chromophores, isolated and aggregated, reveal a fairly good order parameter after relaxation (0.74 and 0.87, respectively). These examples illustrate the complexity of the phase behavior: In most cases (four of six), isolated chromophores are better aligned than those in aggregates. In the two cases, where the aggregates show better alignment than the isolated chromophores, the aggregate alignment is close to ideal, and the difference in the order parameter is very small. Thus, these two analysis results should be regarded as outliers. The model order parameter is often determined by the majority of chromophores, which tend to aggregate in the relaxation step.

Aggregation occurs after long-term relaxation above *T*_g_. The DOA increases from 0.57 to 1.0, because the aggregates are dominated by local interactions while no electric field is present. These local interactions lead to total aggregation, and this impacts the total order parameter, which decreases to a significantly lower value (0.42).

In Table 5, there is no significant difference in the DOA value for the different high concentration models at the different simulation steps (all values ranging between 0.75 and 0.89: ±14% ≈ 4 chromophores). The majority of chromophores are aggregated, and these aggregated chromophores stay together, even at temperatures above *T*_g_ in the course of the long-term relaxation. Compared to the low concentration models, neither total aggregation nor dissolution of aggregates is observed. Each group of chromophores (either isolated or aggregated) shows a notable decrease in the order parameter value (except the group of isolated chromophores of the low order parameter model). In the long term relaxation step, isolated chromophores appear to be more independent of the overall reorientation process, resulting in slightly higher order parameter values compared to the group of aggregated chromophores. On the time scale of the short relaxation, this observation is not as clear as compared to the longer relaxation step (*T* < *T*_g_), presumably representing the better mobility of the isolated chromophores and consequently their faster reorientation compared to the collective reorientation of the larger aggregates. In the low concentration model set, smaller and fewer aggregates are formed compared to those in the high concentration model set. Therefore, aggregated chromophores are almost as movable as isolated chromophores, resulting in varying order parameter values after a short relaxation step, with respect to the order parameter of isolated chromophores.

In conclusion, aggregate size determines the time scale of reorientation. Large aggregates may stabilize the overall alignment, but a good alignment must be achieved beforehand. Isolated chromophores can be easily aligned, but good mobility may result in poor alignment stability. Nevertheless, the relaxation process is temperature dependent and temperatures above *T*_g_ demonstrated a drastically drop of the order parameter value.

## Conclusion and Outlook

In this study, we introduced an automated, flexible, and systematic methodology for analyzing the aggregation and phase behavior of prolate spheroid molecules in condensed matter, such as fluids, polymers, and solids. Our Python-based tool supplies statistical and graphical outputs and is adaptable across different model systems and materials. The analysis method could be applied to different classes of materials (e.g., for optical materials, organic photovoltaics,^12^ organic thin film transistors^29^) where controlled aggregation with­(out) additives or self-organization phenomena (e.g., for liquid crystalline systems)^30,31^ play a crucial role.

We demonstrated the method on dipolar chromophore molecules exhibiting a prolate spheroid (rod-like) shape. However, our method is flexible, and if the shape of the molecule of interest (e.g., oblate/disk-like instead of prolate/rod-like) is taken into account, other shaped molecules and aggregation or self-organization models can be investigated as well. It is important that the reference points are placed on structural elements with a fixed relative orientation. Otherwise changes in molecule conformation may skew the results produced in the analysis procedure. For disk-like chromophores, for example, only the midpoint is important as a characteristic reference point. For surfactants, macromolecules, lipids, etc., characteristic points must be defined along rigid units. The distance criteria have to be adjusted, to those known from either the literature or experiments. Otherwise, these can be determined by MD, as demonstrated in this paper. If there is a differing number of characteristic points compared to the chromophore presented in this article, the functions of the Python script must be adjusted. We provide all Python scripts for individual adaptations or extension.

The presented method is beneficial to gain insights and to point out structure–property relationships. Identification of aggregates enables the visualization of aggregation processes and an a posteriori examination of different aggregates on a higher level of computational theory to exactly quantify^6,9–12^ the EO response of the (controlled) aggregated chromophores is possible.

We identified distinct aggregation classes and size distributions. The tool proved useful for detecting overload concentrations of guest molecules. This is especially important in systems where aggregation is undesirable. Aggregated or purely isolated structure models can be easily distinguished.

Our developed tool provides information on the phase behavior, i.e., the stepwise changes of aggregation in the course of applied simulation protocol(s), which is beneficial to investigate external influences, e.g., temperature dependencies or applied electric fields. Moreover, the output comprises order parameter values of each aggregate, so that cooperative interactions between chromophores become visible. Although our initial study^26^ recommended using a strong electric field to achieve optimal chromophore alignment, future research should aim to identify the minimum field strength necessary for effective alignment within a feasible molecular dynamics time frame. Determining this minimum will enhance experimental comparability, and it remains crucial to investigate whether the chromophore phase behavior is consistent under these adjusted conditions.

The study emphasizes the use of annealing for structure preparation, transforming large chromophore aggregates into more realistic distributions. Using multiple models improved the reliability of our results and the representativeness of molecular modeling studies, especially for amorphous polymers. This approach enabled us to statistically analyze average aggregation sizes and identify frequently occurring or predominantly observed aggregate classes across different model sets, allowing for more general conclusions. We recommend that future studies similarly utilize multiple independent structures to fully capture the variability inherent in amorphous condensed matter.

For future evaluations using our aggregation analysis, the history of the model structure development (e.g., simulation protocol: temperature program, electric field strength, etc.) should always be specified when comparing different models or systems. In the future, the influence of the electric field strength on aggregation and phase behavior should be critically examined in simulations.

Aggregate dynamics are crucial for both the poling efficiency and alignment stability. Future research should explore the optimal aggregate size under specific chromophore concentrations and poling conditions. These studies should aim to achieve efficient poling and enhance alignment stability, particularly at lower field strengths comparable to laboratory settings, thereby impacting material performance, which is directly correlated to optical activity.

## Supplementary Material



## Data Availability

The developed Python script, including a minimal example, the full set of coordinates, and all resulting data tables, is available free of charge at https://gitlab.uni-hannover.de/erik.rohloff/aggregation-analysis/.

## Notes

S1 Description of aggregation analysis script, S2 Supporting data (order parameter values, calculation time in comparison), S3 Figures of all studied systems (PDF)

## §

Passed away on January 13, 2023.
