# Supplementary material for: Aggregation Analysis of Simulated Electric Field Poled Poly(methyl methacrylate) Doped with Tricyanopyrroline Chromophores
Source: J Phys Chem B. 2025 Sep 16;129(38):9804–17. doi: 10.1021/acs.jpcb.5c05224 (PMC12478870; doi:10.1021/acs.jpcb.5c05224)
Supplement: Supplementary file 1 [file jp5c05224_si_001.pdf]

Supporting Information: Aggregation Analysis of Simulated Electric Field Poled Poly(methyl methacrylate) Doped with Tricyanopyrroline Chromophores

Nils M. Denda,[†,‡] Erik Rohloff,[†] Peter Behrens,[†,‡,¶] and Andreas M. Schneider[*,†,‡]

†*Institute of Inorganic Chemistry, Leibniz University Hannover, 30167 Hannover, Germany*

‡*Cluster of Excellence PhoenixD (Photonics, Optics, and Engineering – Innovation Across Disciplines), 30167 Hannover, Germany*

¶*Passed away on January 13, 2023*

E-mail: andreas.schneider@acb.uni-hannover.de

Phone: +49 (0) 511 762 3259. Fax: +49 (0) 511 762 3006

Contents

S1 Description of Aggregation Analysis Script **S2**

S2 Supporting Data **S4**

S2.1 Order Parameter Values of All Models S4

S2.2 Order Parameter Values of a Selected Few Special Models S4

S2.3 Calculation Time in Comparison S5

S3 Figures of All Studied Systems **S6**

S1 Description of Aggregation Analysis Script

The aggregation analysis tool is available at https://gitlab.uni-hannover.de/erik.rohloff/aggregation-analysis/.

1. **Input parsing:**
   - Input file:
     - Edge length of simulation box (to account for periodic boundary conditions)
     - Center of geometry coordinates (mid points)
     - Head and tail coordinates
   - Calculation of individual order parameter for each chromophore
2. **Definition of aggregation and detection:**
   - Specification of midpoint-to-midpoint (MM) and head-to-tail (HT) distances
   - Separation distances evaluation for each chromophore pair
   - Identification of interrelated chromophores with union-find algorithm
3. **Aggregate analysis:** Within each identified aggregate:
   - Classification of pairwise interactions between chromophores into
     $\rightarrow$ isolated (i), stack-like (s) or chain-like (c) interactions
   - Accumulation of (i/s/c) interaction counts
   - Attribution of dominant aggregation mode for each aggregate:
     $\rightarrow$ Stack- or chain-like
     $\rightarrow$ Mixed when neither interaction is significantly more present
   - Order parameter calculation of each aggregate + average over all aggregates
   - Average order parameter calculation of all isolated chromophores

(Continued on the next page.)

4. **Output files:**

- summary .txt file: cumulated summary of in- and output, i.e.:
  - Model name(s)
  - Chromophore number: mid point coordinates (x, y, z)
  - Aggregate counts
  - Aggregate compositions + individual order parameter values
  - Average order parameter of isolated chromophores
  - Model order parameter
- data .csv files for bar plots:
  - Aggregate class distribution
  - Aggregate size distribution
  - Aggregate-class-size distribution
  - Model set average plots

S2 Supporting Data

S2.1 Order Parameter Values of All Models

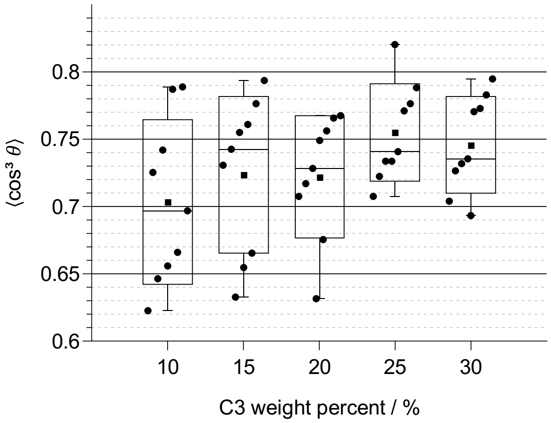

Figure S1: All order parameter values $\langle \cos^3 \theta \rangle$ plotted in a box plot. Squares showing average values and the box represents the standard deviation.

S2.2 Order Parameter Values of a Selected Few Special Models

Table S1: Models with minimum (min) / median (med) / maximum (max) order parameter values $\langle \cos^3 \theta \rangle$ after relaxation (averaged over 2 ns, all standard deviations are below 0.03).

Model set	$\langle \cos^3 \theta \rangle$ min … med … max
10 wt%	0.62 … 0.70 … 0.79
20 wt%	0.62 … 0.73 … 0.77
30 wt%	0.69 … 0.74 … 0.79

S2.3 Calculation Time in Comparison

The standard poling and relaxation protocol consists of 50 ns total simulation time (t_{MD}), which requires approx. 113 h (= 4.7 d) calculation time (t_{CPU}). The long-term relaxation is set to 100 ns (instead of 20 ns), the total simulation time is increased to 130 ns and requires approx. 284 h calculation time (= 11.8 d). These estimates are based on one computing machine: Intel Xeon Gold 6252 CPU, 2.10 GHz (Dual). 16 CPU cores have been utilized for the calculation.

S3 Figures of All Studied Systems

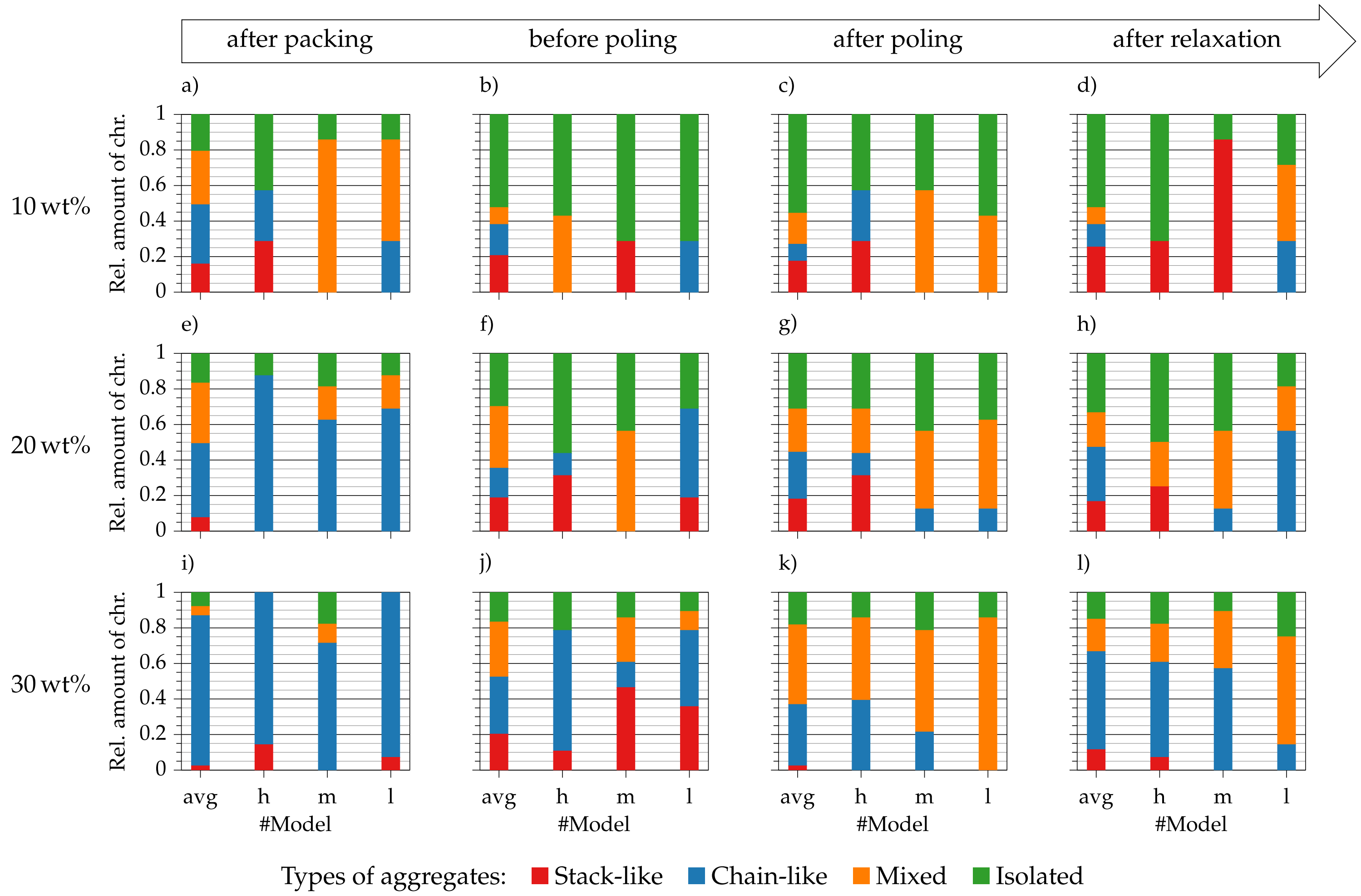

Figure S2: Classification of chromophore aggregates in the course of the employed simulation protocol (horizontal direction: a) - d) / e) - h) / i) - l)) dependent on the C3 mass percentage 10 wt% to 30 wt% (vertical direction, “model set” = nine models). Every first bar represents the average phase behavior of all nine models in a given model set. The following bars are chosen models from this set according to the order parameter value $\langle \cos^3 \theta \rangle$ after relaxation (“h” = model with highest $\langle \cos^3 \theta \rangle$, “l” = model with lowest $\langle \cos^3 \theta \rangle$, “m” = model with median $\langle \cos^3 \theta \rangle$).

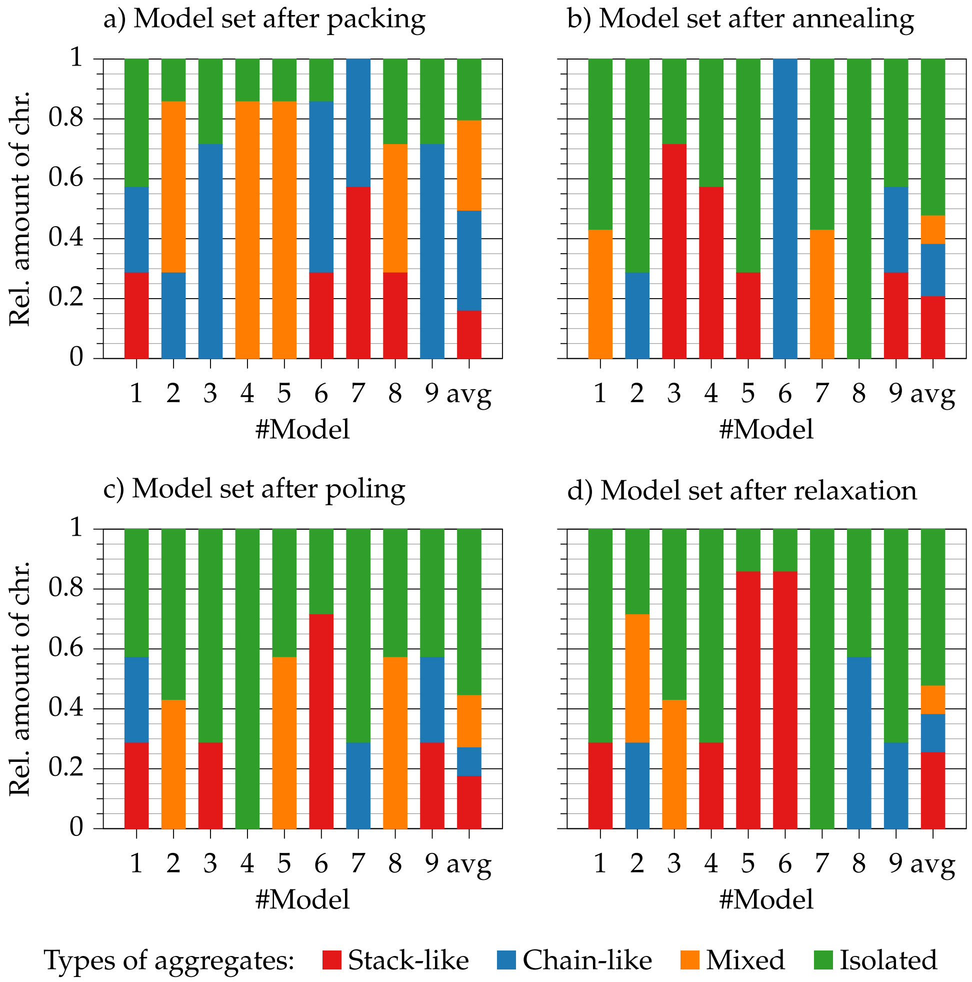

Figure S3: Aggregation analysis on all 10 wt% models in the course of the poling and relaxation program a) - d).

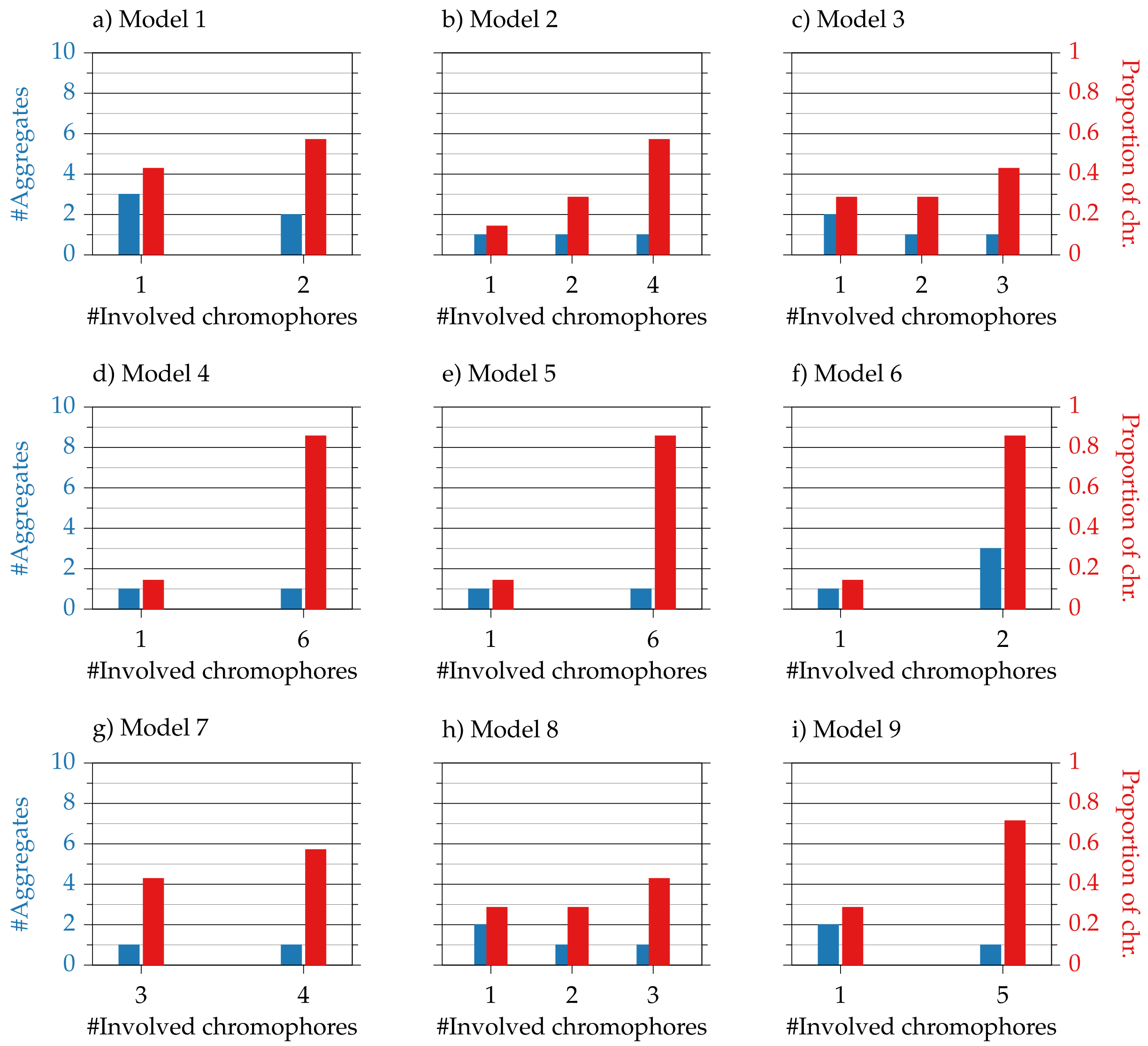

Figure S4: Composition of observed aggregates in models with 10 wt% C3 after packing.

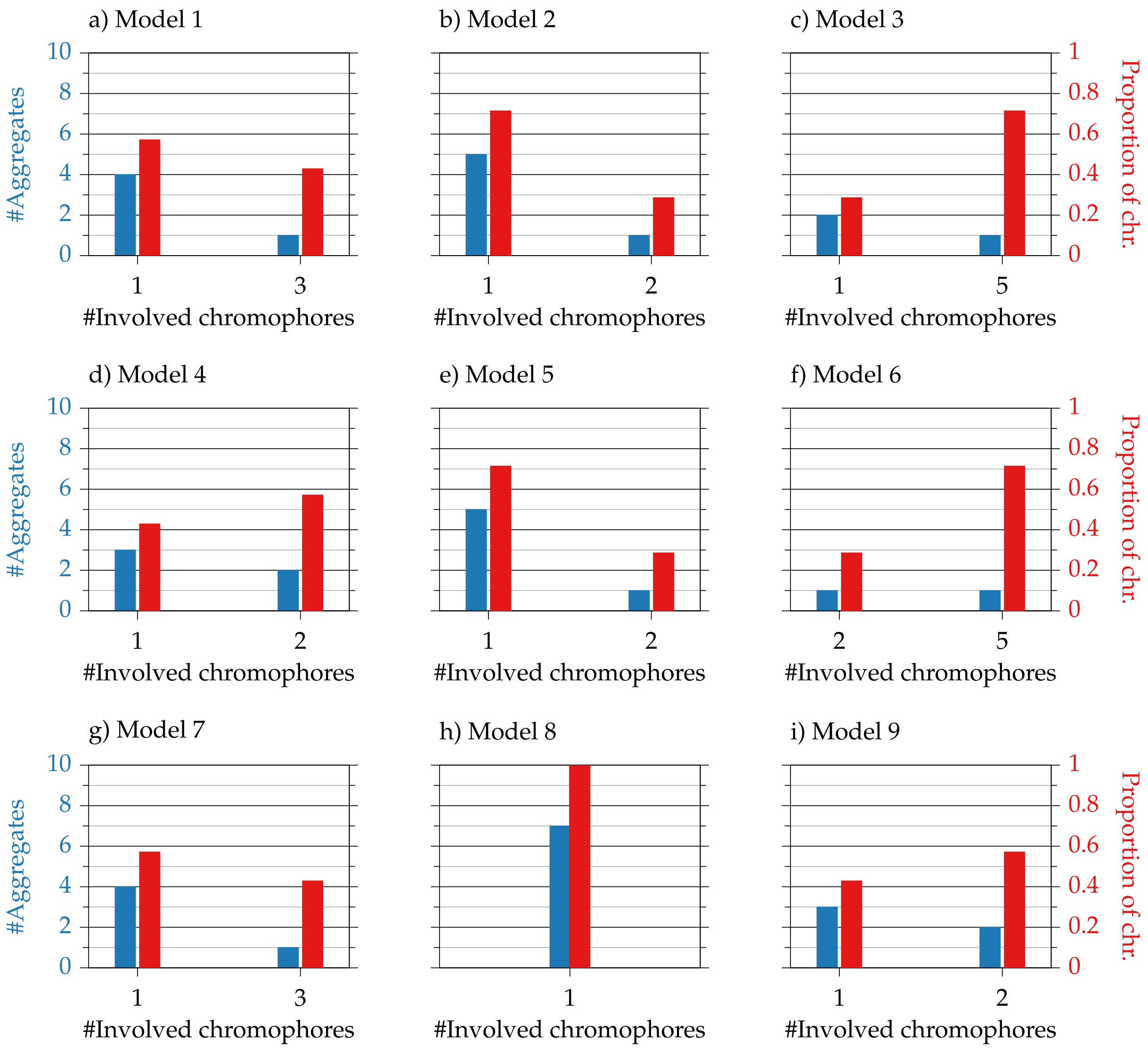

Figure S5: Composition of observed aggregates in models with 10 wt% C3 after annealing.

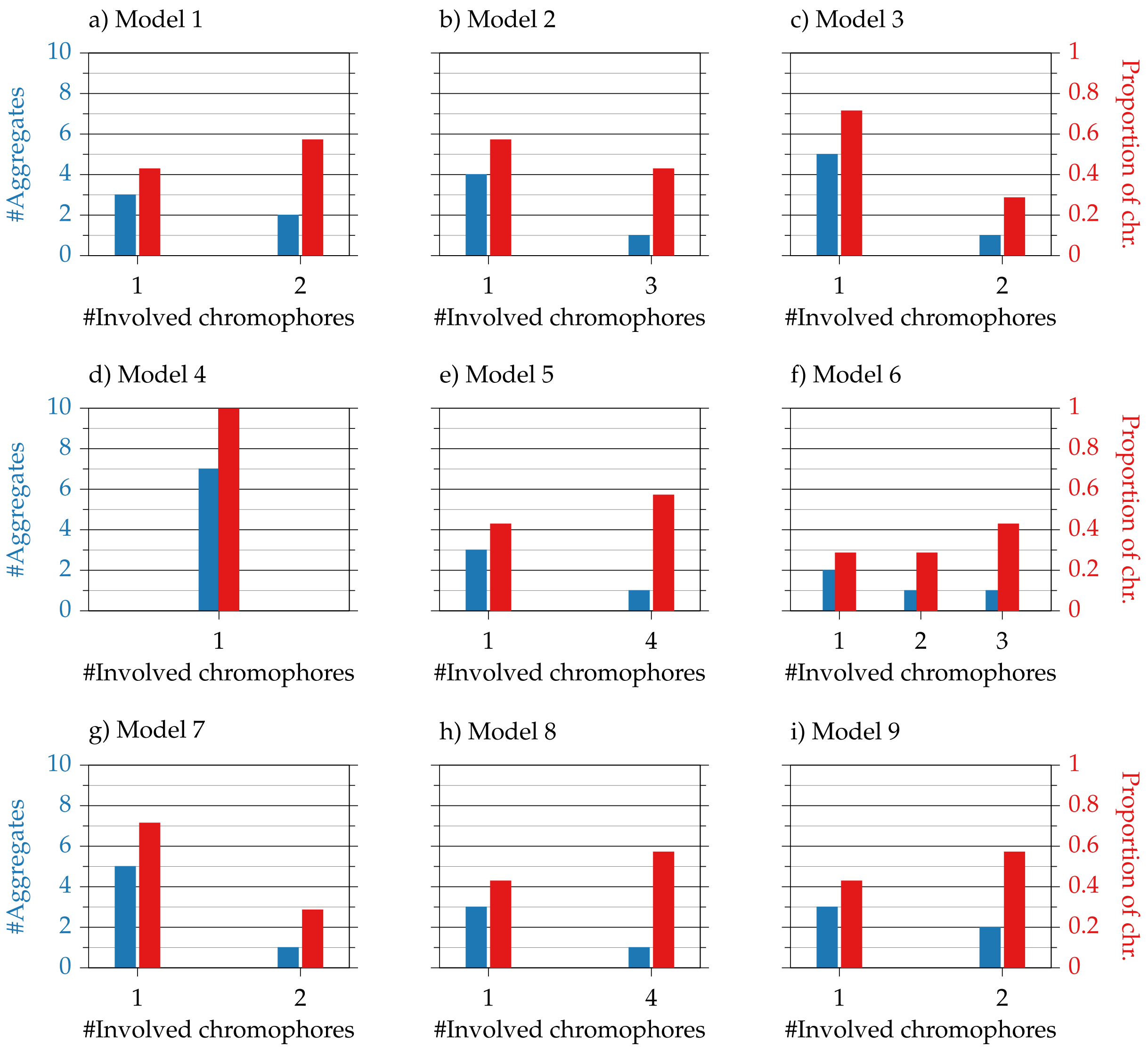

Figure S6: Composition of observed aggregates in models with 10 wt% C3 after poling.

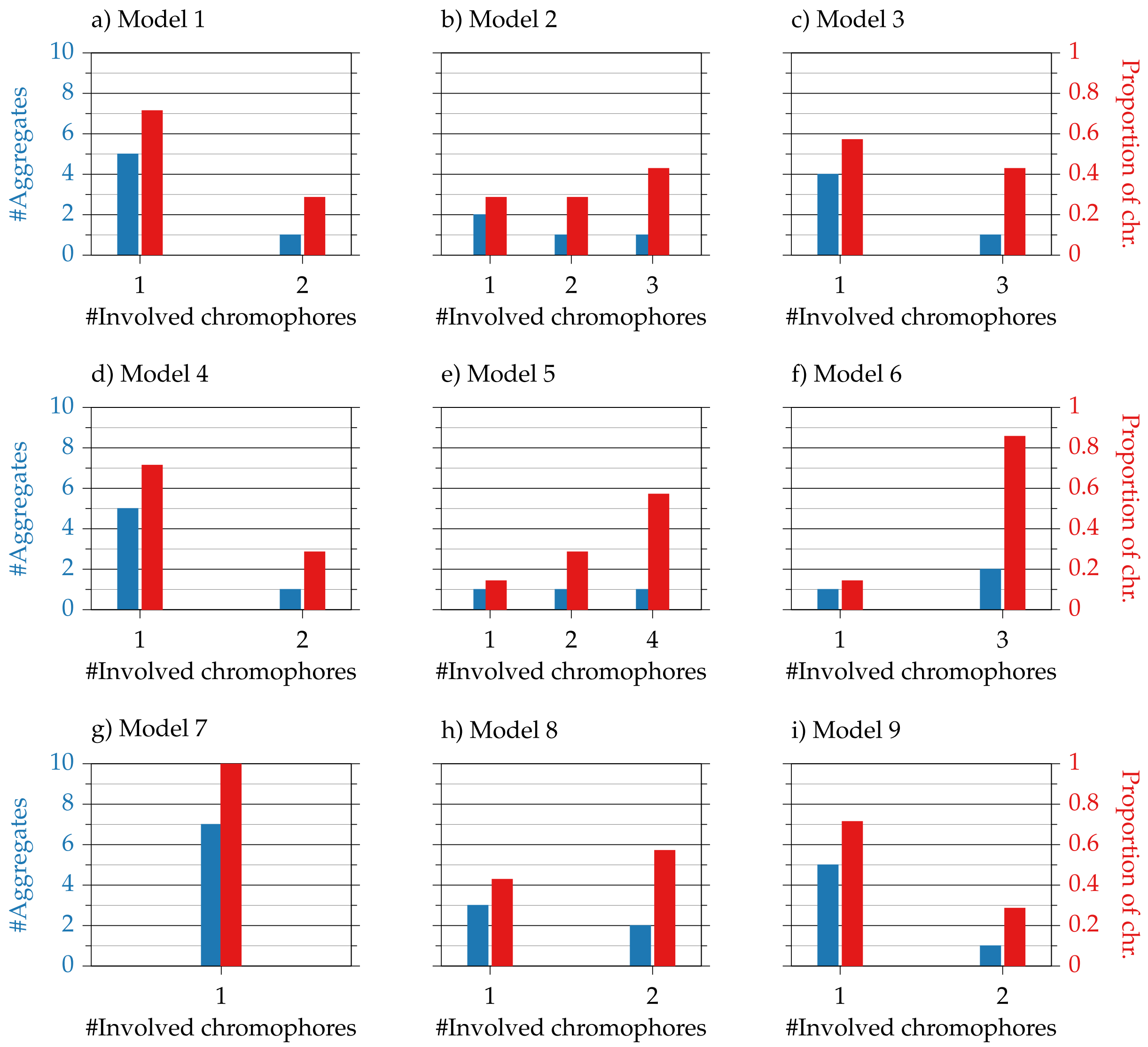

Figure S7: Composition of observed aggregates in models with 10 wt% C3 after relaxation.

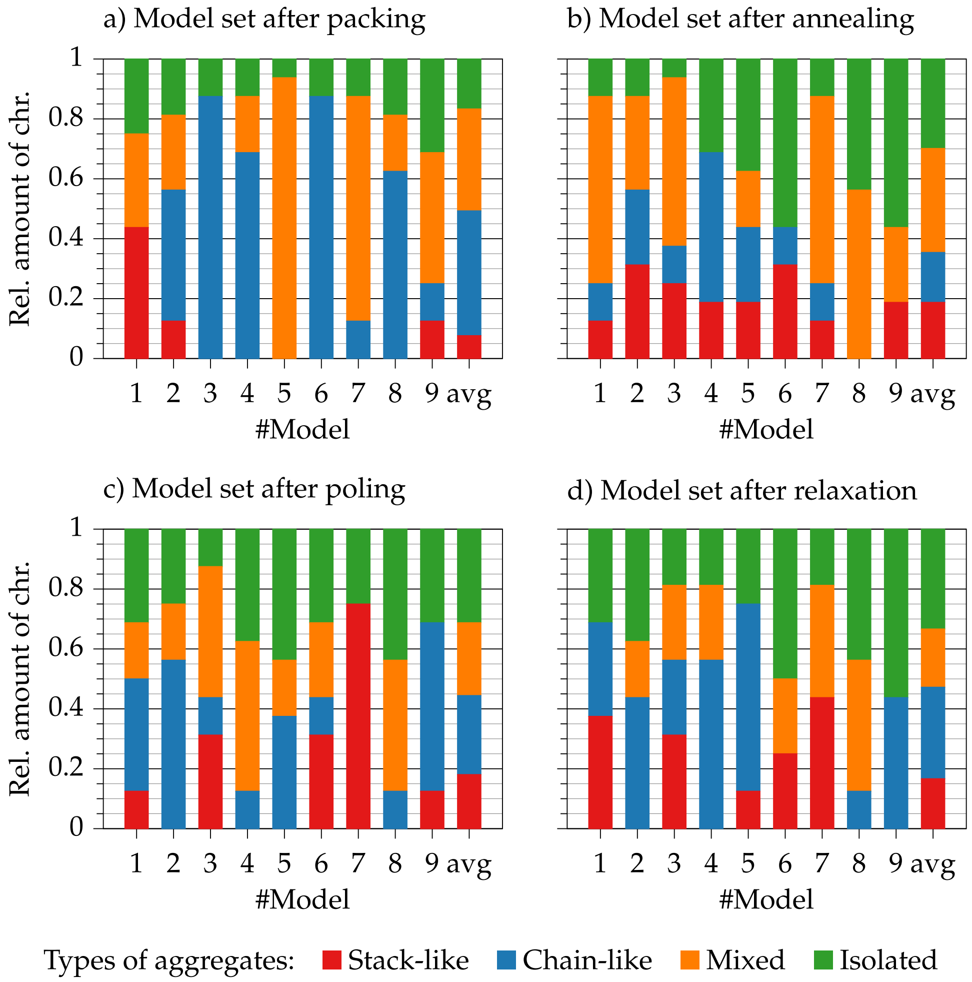

Figure S8: Aggregation analysis on all 20 wt% models in the course of the poling and relaxation program a) - d).

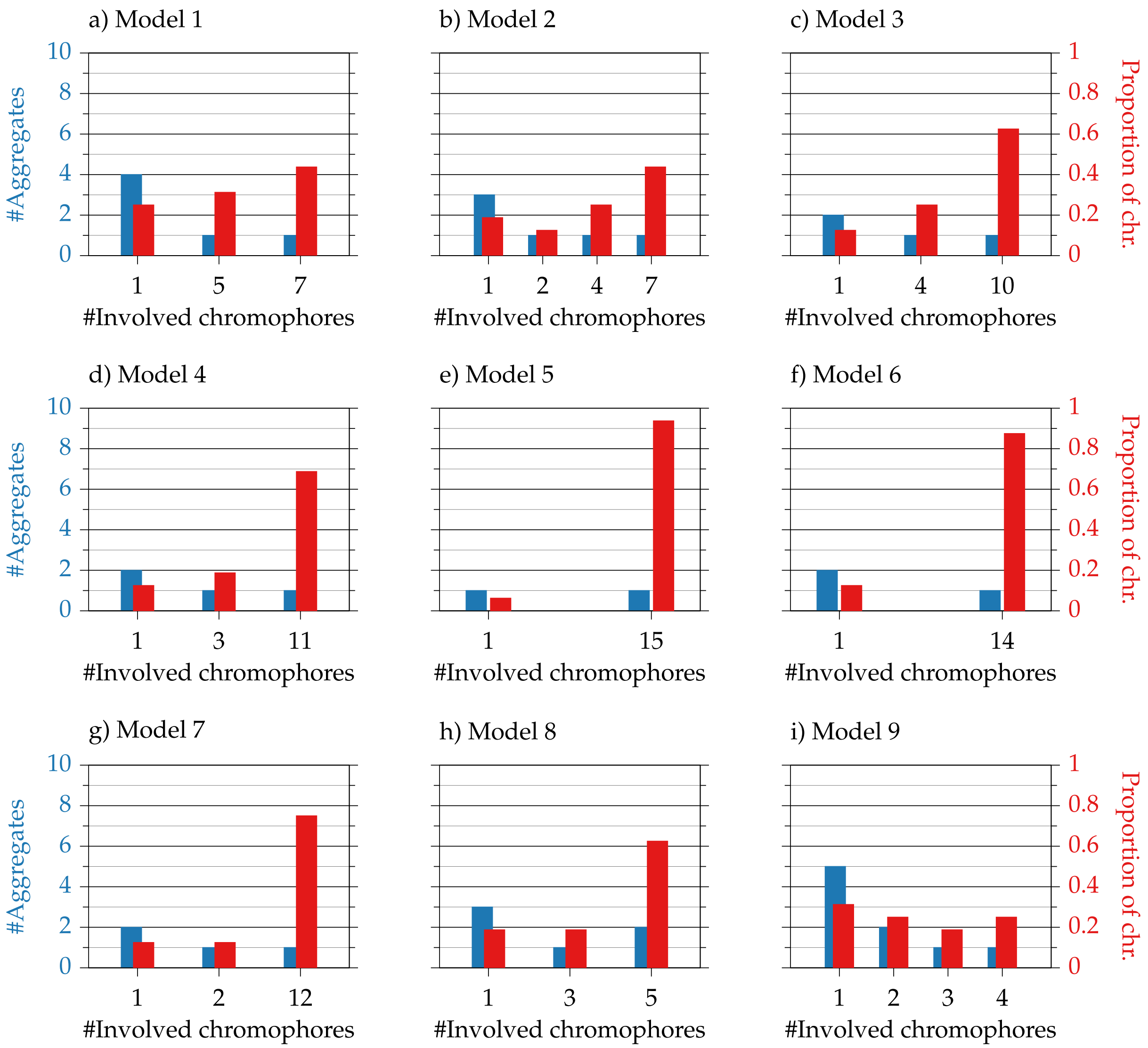

Figure S9: Composition of observed aggregates in models with 20 wt% C3 after packing.

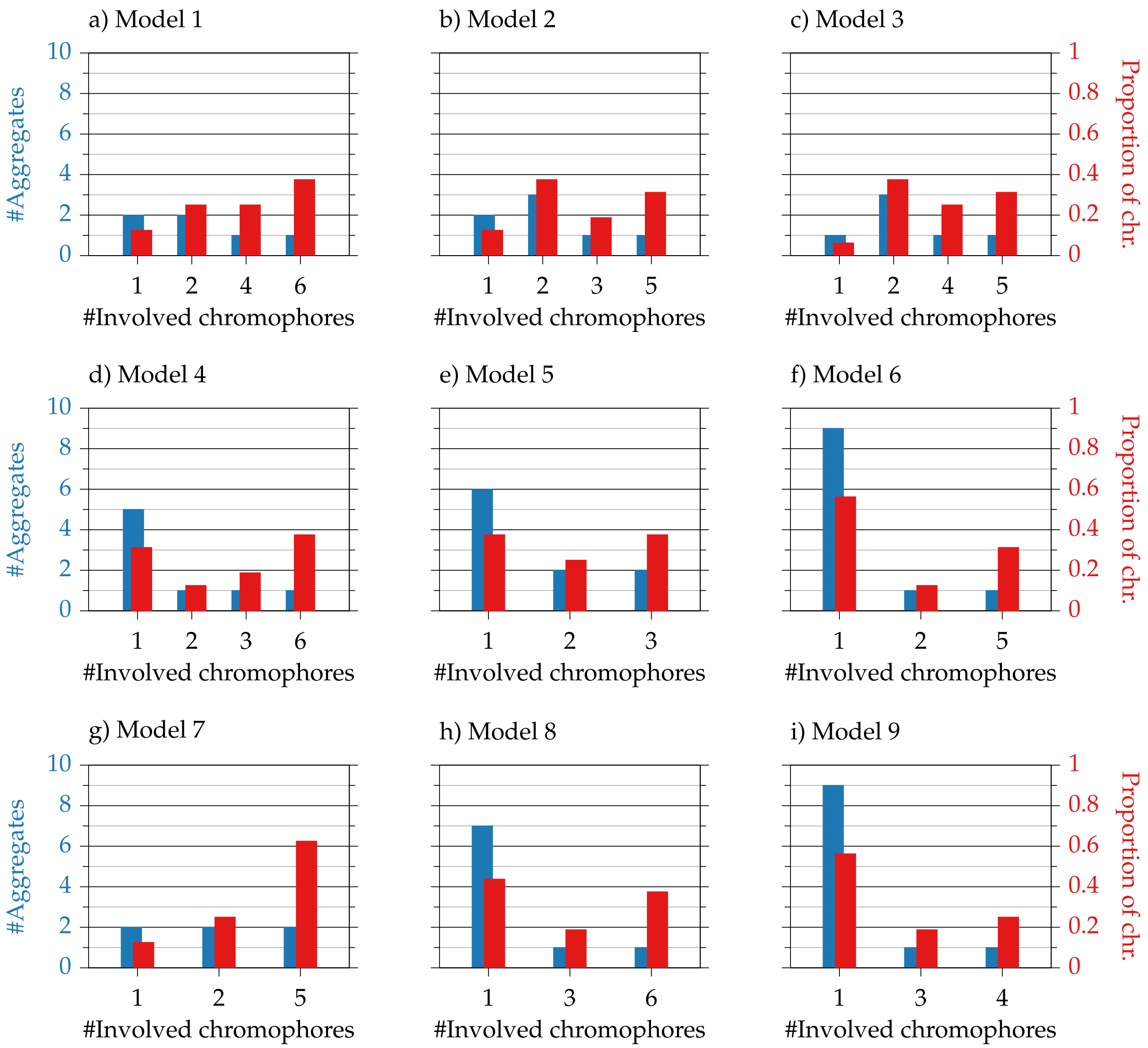

Figure S10: Composition of observed aggregates in models with 20 wt% C3 after annealing.

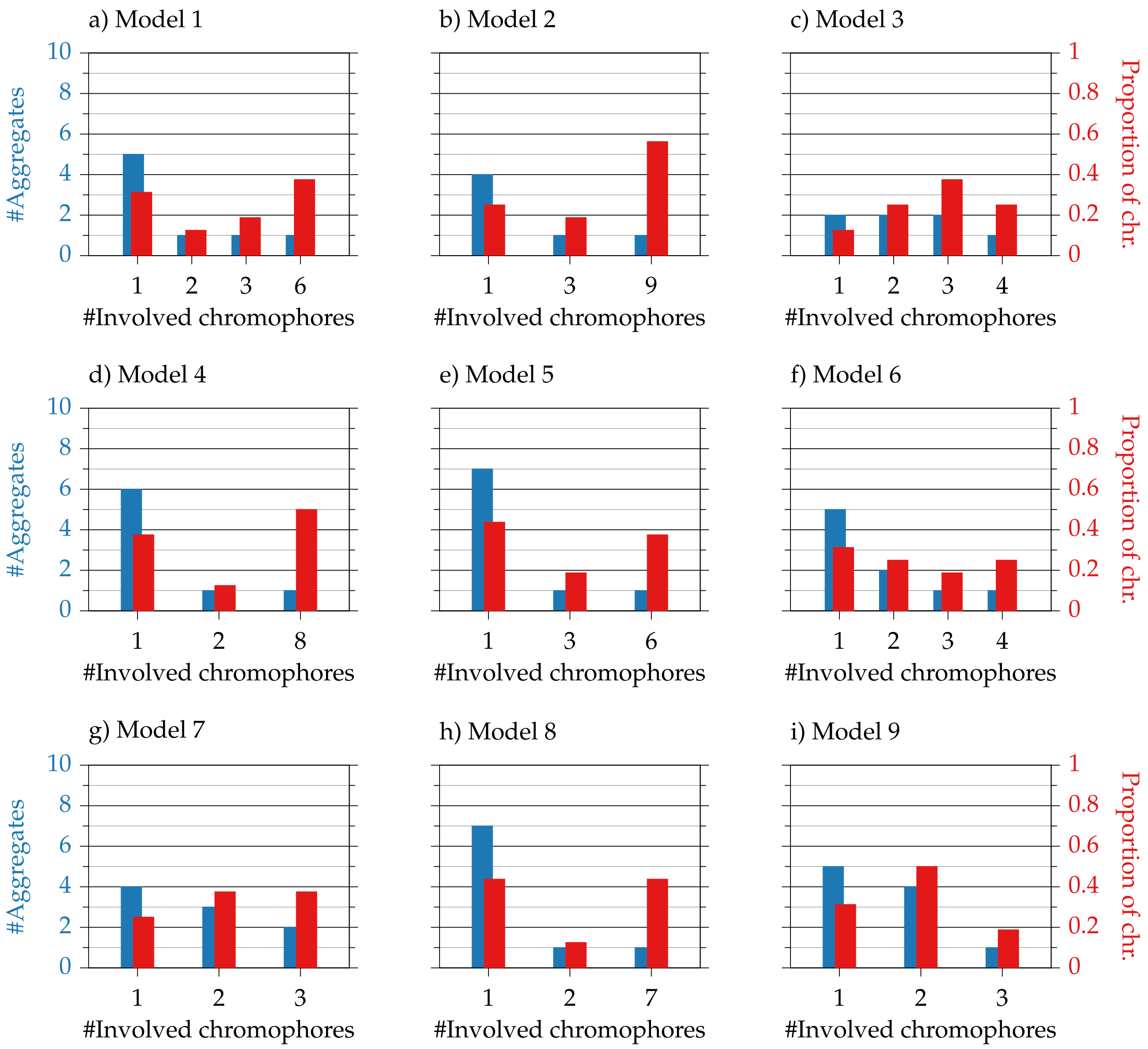

Figure S11: Composition of observed aggregates in models with 20 wt% C3 after poling.

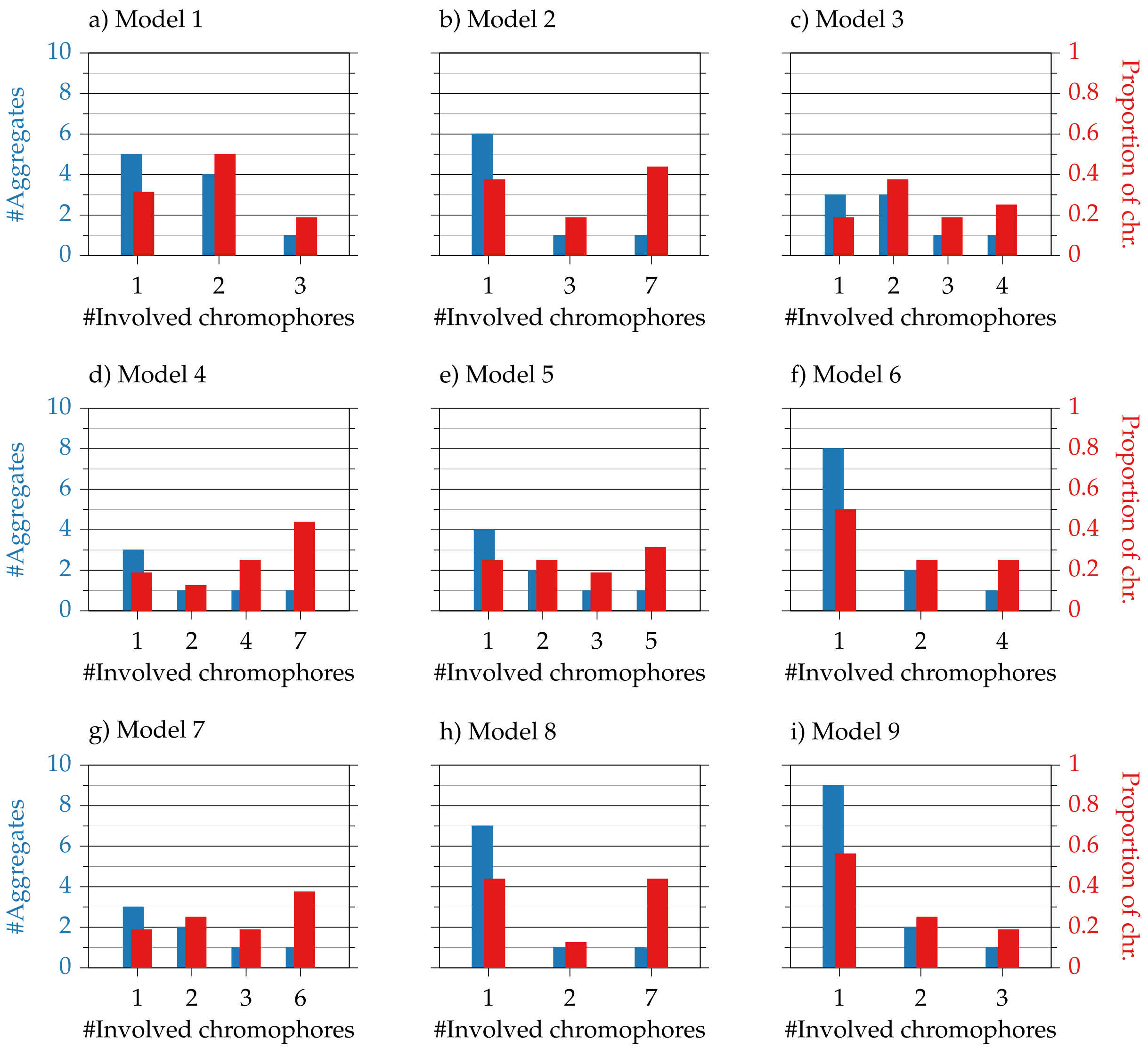

Figure S12: Composition of observed aggregates in models with 20 wt% C3 after relaxation.

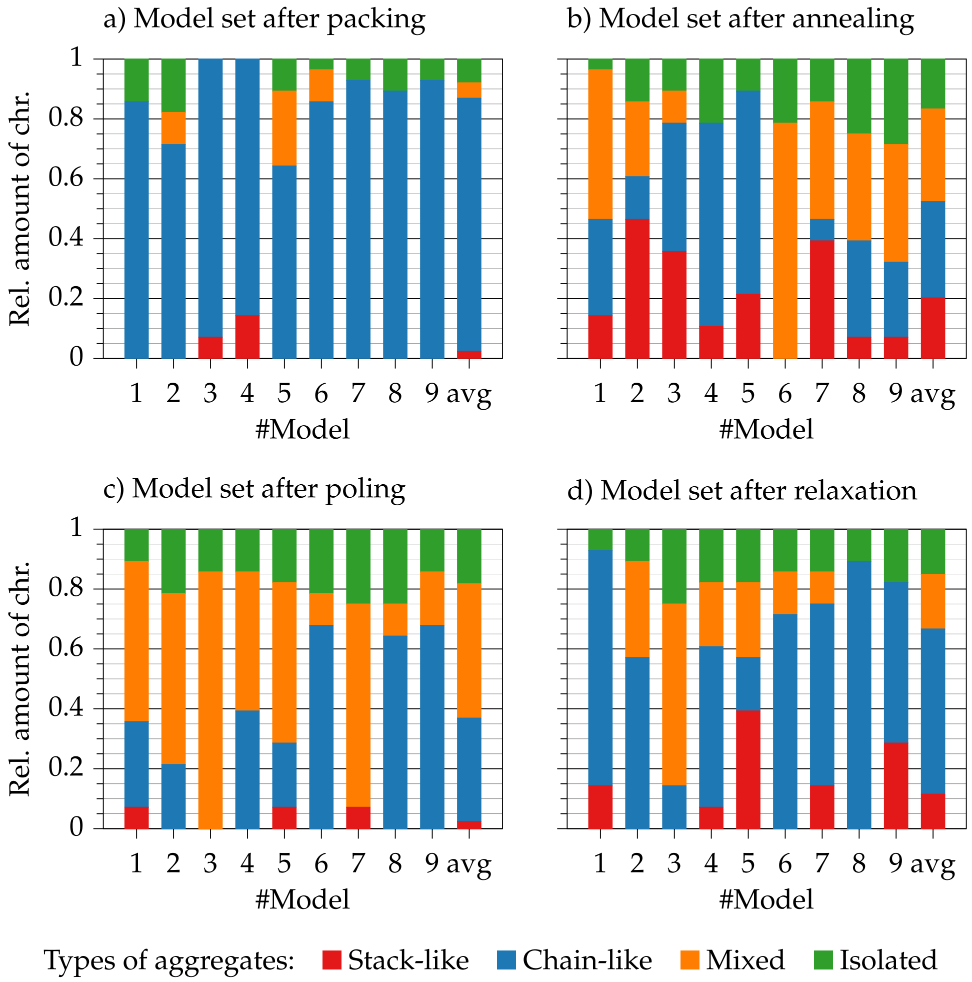

Figure S13: Aggregation analysis on all 30 wt% models in the course of the poling and relaxation program a) - d).

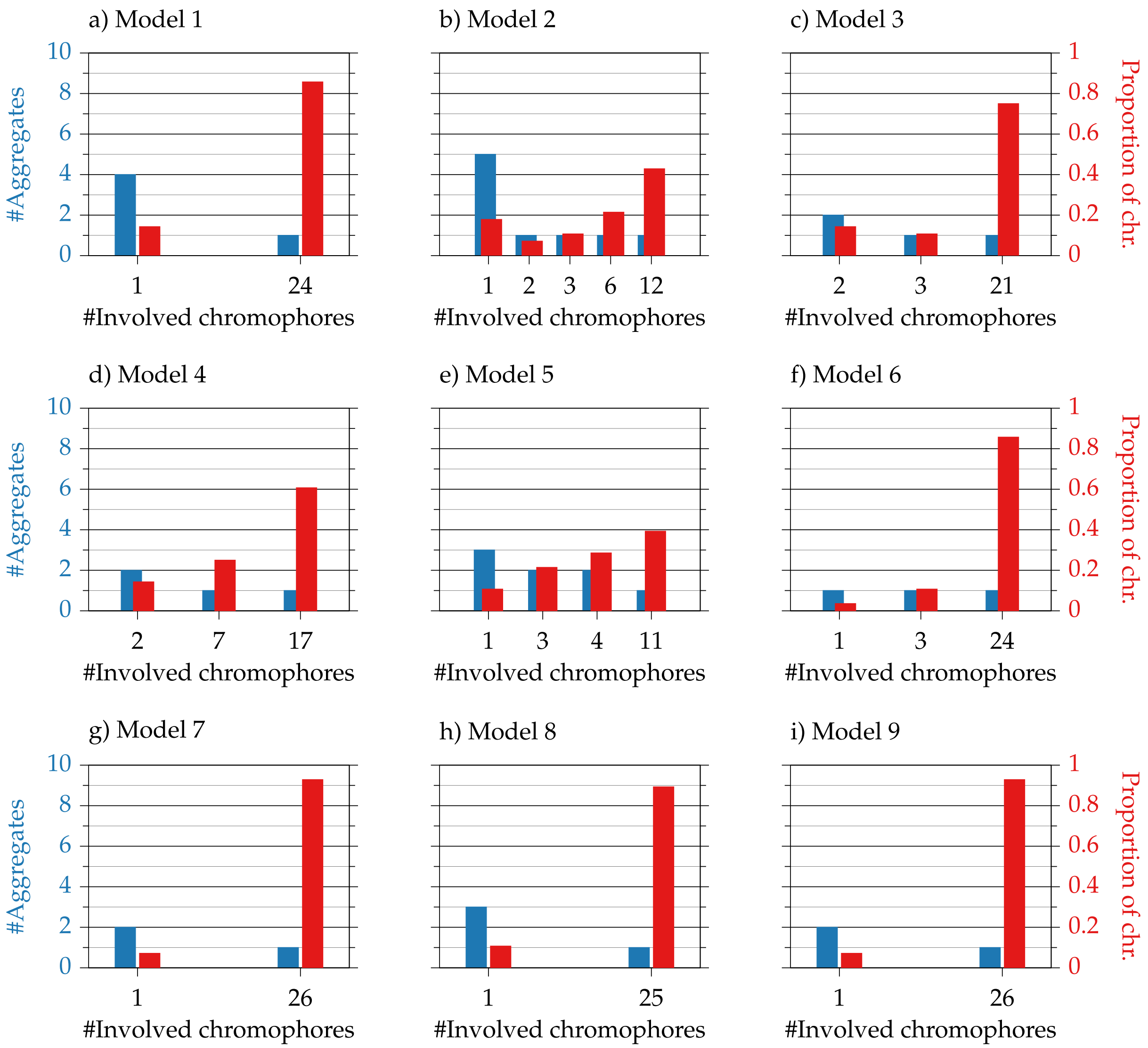

Figure S14: Composition of observed aggregates in models with 30 wt% C3 after packing.

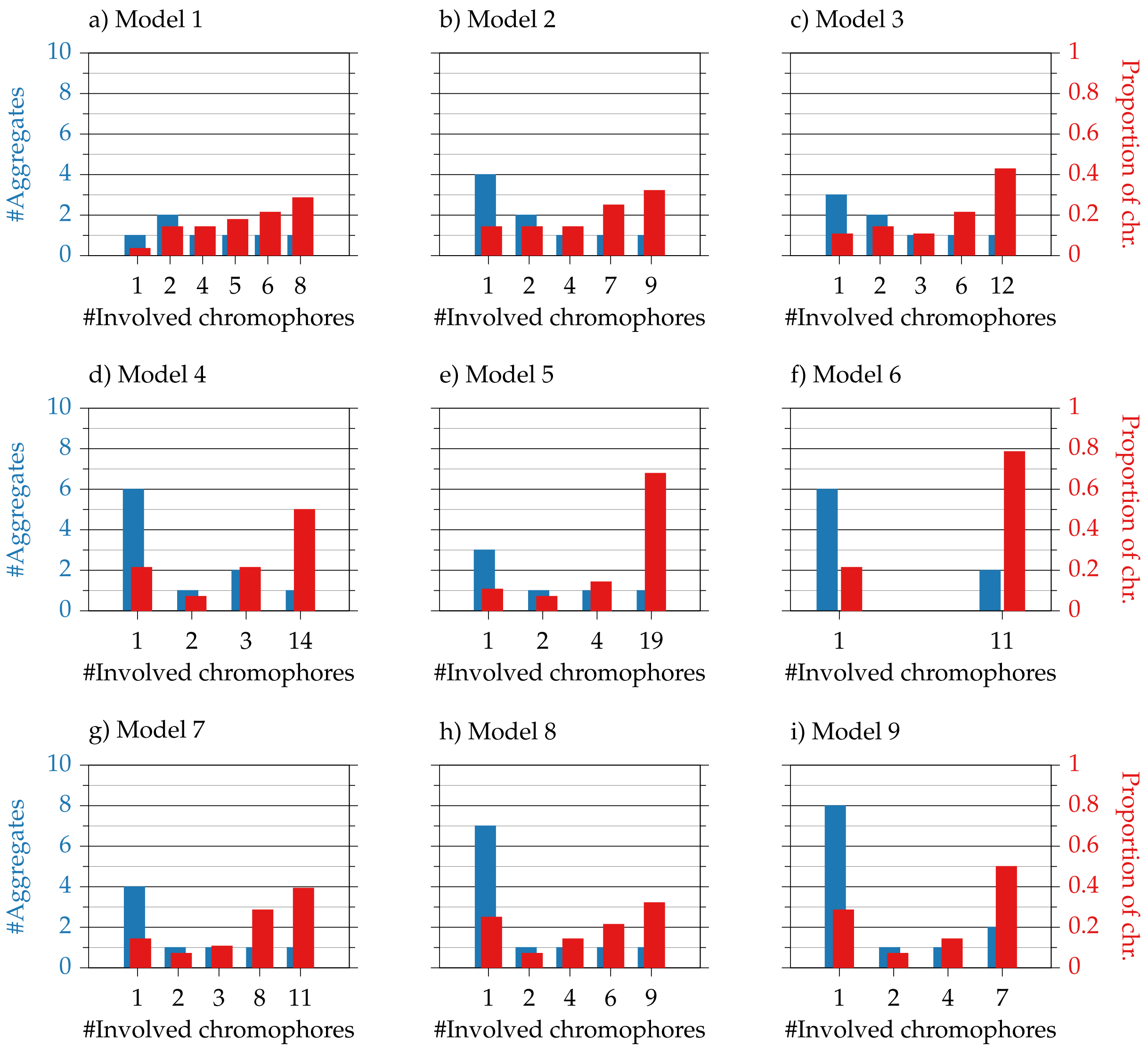

Figure S15: Composition of observed aggregates in models with 30 wt% C3 after annealing.

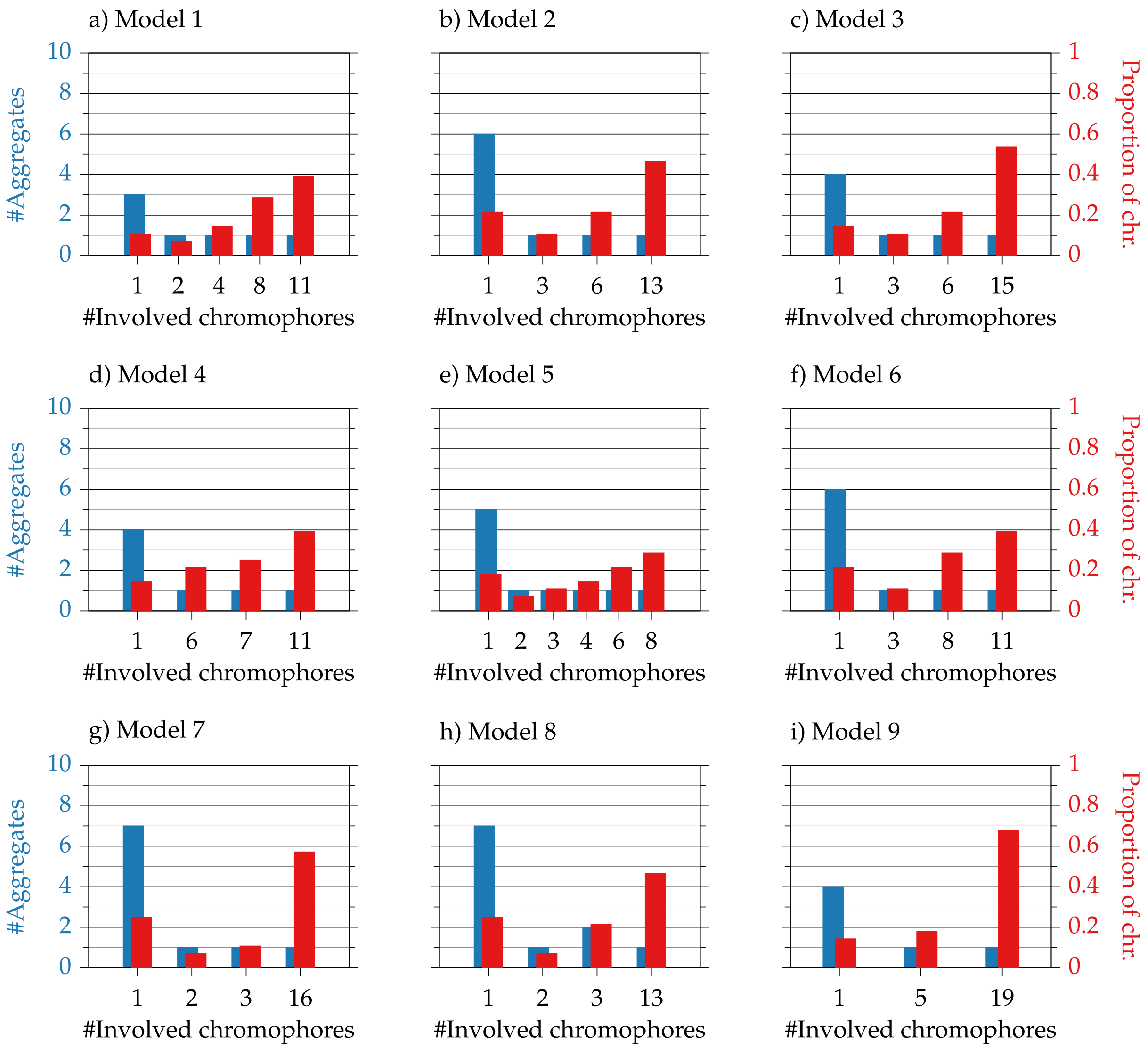

Figure S16: Composition of observed aggregates in models with 30 wt% C3 after poling.

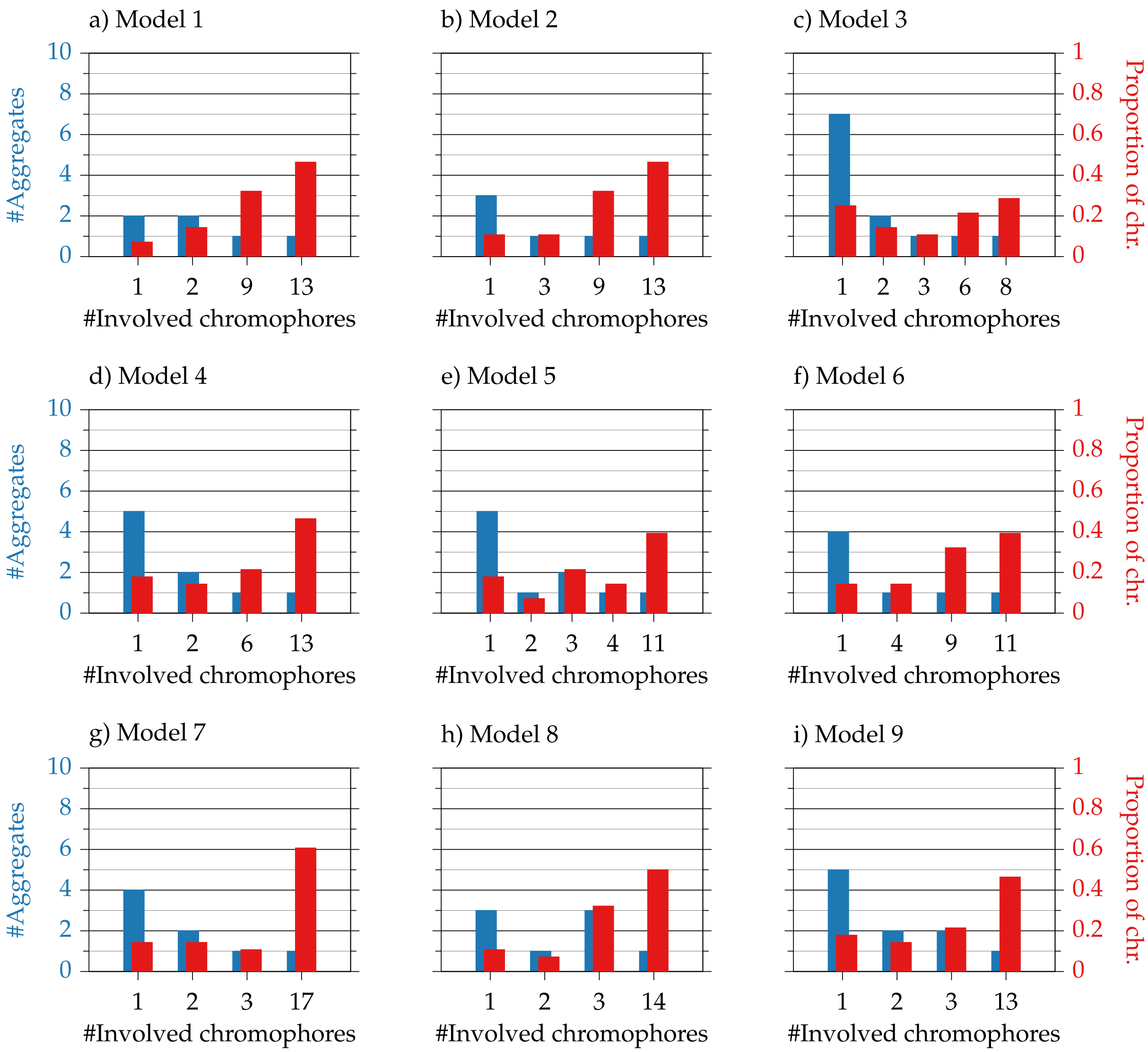

Figure S17: Composition of observed aggregates in models with 30 wt% C3 after relaxation.
